# Salt intake moderates the association between BMI and cardiometabolic risk: evidence from an occupational cohort in Beijing

**DOI:** 10.3389/fnut.2026.1815741

**Published:** 2026-04-22

**Authors:** Jiaju Ren, Yanqing Guo, Yanbo Zhu, Yan Cui, Li Jin, Wei Hao, Yanju Zhang

**Affiliations:** 1 School of Management, Beijing University of Chinese Medicine, Beijing, China; 2 School of Traditional Chinese Medicine, Beijing University of Chinese Medicine, Beijing, China; 3 Health Management Department, Beijing Genertec Aerospace Hospital, Beijing, China

**Keywords:** body mass index, cardiometabolic indicators, daily salt intake, Johnson-Neyman analysis, restricted cubic splines

## Abstract

**Objective:**

This study aims to examine whether daily salt intake (DSI) modifies the association between body mass index (BMI) and cardiometabolic indicators in a Beijing occupational population.

**Methods:**

In this cross-sectional study, 1,866 adults undergoing occupational health examinations were included. DSI was estimated from fasting morning spot urine using the Sun_C equation. Based on correlation screening, seven cardiometabolic indicators were selected for further analysis: HDL-C, uric acid (UA), ApoA1, creatinine (Cr), ALT, ApoB/ApoA1, and diastolic blood pressure (DBP). Associations were assessed using multiple linear regression and restricted cubic spline models adjusted for age and sex. Effect modification by DSI was evaluated using interaction terms and the Johnson–Neyman technique.

**Results:**

Mean DSI was 9.50 ± 2.18 g. RCS analysis revealed significant non-linear associations between BMI and markers, including HDL-C, ApoA1, and UA (all P-non-linear < 0.05), with inflection points concentrated at 24.95 kg/m^2^. DSI significantly moderated the relationships between BMI and HDL-C and ApoA1, whereas the interaction for UA was suggestive but did not reach statistical significance. In the young and middle-aged population (<60 years), these effects followed distinct sex-specific threshold patterns. Specifically, the lower DSI moderating thresholds for men ranged from 13.4 g to 16.2 g (encompassing HDL-C, UA, and ApoA1), while thresholds for women were notably lower, ranging between 11.2 g and 12.4 g. When salt intake remained below these limits, the adverse effects of obesity on metabolic indicators were significant and robust. Among elderly participants, all interaction terms were non-significant (*p* > 0.2).

**Conclusion:**

The moderating influence of daily salt intake on cardiometabolic associations with BMI varied by age and was more evident in younger adults. The thresholds identified in this study differed by sex, at approximately 13–16 g/d in men and 11–12 g/d in women, and were higher than the WHO-recommended ceiling of 5 g/d. In practical terms, although most participants remained below these sex-specific thresholds, the metabolic burden of excess body weight was evident across the working-age adult population regardless of current sodium intake. These findings suggest that a single sodium restriction target may not fully reflect differences in metabolic context, although the present results should be interpreted cautiously in light of the cross-sectional design.

## 1 Introduction

Managing dietary sodium intake has long been both a consensus and a point of debate in public health research (1–3). A large body of epidemiological and experimental evidence has established that excessive sodium intake elevates blood pressure and increases cardiovascular risk. On this basis, the World Health Organization and many national health authorities, including those in China, recommend limiting daily salt intake in adults to no more than 5–6 g (4–9). These recommendations stem mainly from the observed associations between sodium consumption, elevated blood pressure, and a higher risk of cardiovascular disease. Excessive salt intake not only raises blood pressure but is also linked to endothelial dysfunction, arterial stiffness, and ventricular hypertrophy, all of which contribute to cardiovascular pathology (10–14).

In practice, sodium consumption remains well above recommended levels in most populations. In China, for example, the average daily intake among adults reaches 10–12 g (15), far exceeding the suggested upper limit. Emerging evidence has also indicated that the relationship between sodium intake and cardiovascular outcomes may not be strictly linear. Several studies have described a J-shaped or U-shaped pattern, suggesting that extremely low sodium intake might also increase cardiovascular risk (16–19). Individual responses to sodium vary widely, influenced by age, sex, ethnicity, and obesity status (20–22). These differences suggest that the application of universal dietary guidelines may not fully capture the diversity of metabolic responses across different individuals. It remains uncertain whether the metabolic correlates of sodium intake may differ across subgroups.

Among various metabolic factors, obesity plays a particularly important role. Body mass index (BMI), a common indicator of obesity, is closely related to metabolic abnormalities such as dyslipidemia, hyperuricemia, and insulin resistance (23). It is therefore plausible that obesity may influence the body’s metabolic response to dietary sodium. The effect of sodium intake on cardiometabolic markers may vary depending on BMI level. Most previous studies have examined sodium intake and obesity as independent or additive risk factors. Few have treated sodium intake as a moderating factor that alters the strength or direction of the relationship between obesity and cardiometabolic indicators across different BMI levels. Adopting such a moderation perspective is essential for understanding the individual variability in the health effects of sodium.

Traditionally, moderation has been tested using the simple slope method, which assesses the significance of the interaction term but cannot determine at which values of the moderator the main effect becomes significant. The Johnson–Neyman (J–N) technique provides a more precise approach by identifying the specific range of the moderator where the conditional effect of the independent variable on the dependent variable changes meaningfully (24). Although the J–N method has been widely used in psychology and education, its application in nutritional epidemiology remains limited. Moreover, the association between BMI and cardiometabolic outcomes may not follow a simple linear pattern. Restricted cubic spline (RCS) models allow for flexible modeling of non-linear dose–response relationships and can test whether non-linearity is statistically significant. This method has been widely used to explore how BMI and other factors relate to a variety of health outcomes (25, 26).

In this study, we identified key cardiometabolic indicators closely associated with daily salt intake (DSI). We then examined whether DSI moderates the relationship between BMI and these indicators in an occupational cohort in Beijing, using the Johnson-Neyman technique to identify the range in which the association changes. Analyses by age and sex were also conducted to explore possible heterogeneity in these patterns.

## 2 Materials and methods

### 2.1 Study design and participants

This cross-sectional study drew participants from routine health examinations at a regional aerospace institution, initially screening 2,936 adults during May to July 2025. Inclusion criteria were as follows: (1) age ≥18 years; (2) completion of the full physical examination and laboratory testing; (3) completion of the dietary questionnaire. Exclusion criteria included those as follows: (1) prior diagnosis of cardiovascular diseases such as coronary heart disease, stroke, or heart failure; (2) severe hepatic insufficiency (ALT >3 × upper limit of normal) or renal insufficiency (eGFR <60 mL/min/1.73 m^2^); (3) history of malignant tumors; (4) use of lipid-lowering agents, uric acid-lowering agents, or diuretics in the preceding 3 months; (5) pregnant or lactating women; (6) missing data or clearly implausible values. Following application of these criteria, 1,866 participants were included. All participants gave written informed consent, and the study protocol was approved by the Research Ethics Committee of the Beijing Genertec Aerospace Hospital (approval number: 2025-0302-01).

### 2.2 Anthropometric measurements

Height was measured with a stadiometer (accurate to 0.1 cm) with participants barefoot, without headwear, standing erect. Weight was measured using calibrated electronic scales (accurate to 0.1 kg) with participants wearing light clothing. BMI was calculated as weight (kg) divided by height (m) squared. Waist circumference was measured at the midpoint between the lower rib margin and iliac crest, hip circumference at the most prominent buttock point, both accurate to 0.1 cm. Waist-to-hip ratio (WHR) was calculated as waist circumference divided by hip circumference.

Blood pressure was measured with certified electronic sphygmomanometers after participants rested quietly for 5 min. The non-dominant arm was used, with the cuff placed 2–3 cm above the elbow crease. Three measurements at 2-min intervals were taken, with the average of the last two recorded as systolic blood pressure (SBP) and diastolic blood pressure (DBP).

### 2.3 Laboratory assessments

Following an 8- to 12-h overnight fast, venous blood samples were collected from all participants in the morning. After centrifugation, the samples were analyzed in a certified clinical laboratory using automated biochemical analyzers. Measured parameters included fasting blood glucose (GLU, assessed by the glucose oxidase method) and a full lipid profile comprising total cholesterol (TC), triglycerides (TG), high-density lipoprotein cholesterol (HDL-C), and low-density lipoprotein cholesterol (LDL-C), all determined by enzymatic methods. Additional analyses encompassed apolipoprotein A1 (ApoA1), apolipoprotein B (ApoB), liver function markers [alanine aminotransferase (ALT), aspartate aminotransferase (AST), and *γ*-glutamyl transferase (γ-GT)], and renal function markers [serum creatinine (Cr) and uric acid (UA)]. The urinary albumin-to-creatinine ratio (ACR) was calculated from morning spot urine samples. Quality control was maintained with intra- and inter-assay coefficients of variation below 5% for all measurements.

### 2.4 Twenty four hour urine sodium measurement and daily salt intake assessment

Daily salt intake (DSI) was estimated from spot urine samples by first deriving 24-h urinary sodium excretion (UNa24h) and then converting it to daily salt consumption. Estimation of 24-h urinary sodium excretion from spot urine is a well-established approach in epidemiological studies when direct 24-h urine collection is not feasible. Several predictive equations have been proposed for this purpose, including the Kawasaki, Tanaka, and INTERSALT equations, which have been widely applied in different populations (27–29). In the present study, UNa24h was estimated using the Sun_C equation, as it was developed and validated in Chinese populations and was therefore considered more suitable for our study sample (30, 31). A single fasting morning urine sample was collected from each participant, and urinary sodium (UNa) and creatinine (UCr) concentrations were measured. The Sun_C equations were as follows:


Male=461.11×(UNaspot(mmol/L)/UCrspot(μmol/L))^0.5+41.14–0.35×Age(years)+0.64×Weight(kg)+0.31×UNaspot(mmol/L)

Female=639.14×(UNaspot(mmol/L)/UCrspot(μmol/L))^0.5–9.42−0.33×Age(years)+1.06×Weight(kg)+0.13×UNaspot(mmol/L)

Daily salt intake is calculated based on 24-h urinary sodium excretion using the formula:


$$\begin{array}{c} \text{Daily salt intake}\;(g)=24-h\;\text{urinary sodium excretion}\;(\text{mmol})\\ \times 0.0585 \end{array}$$

Here, 0.0585 is the conversion factor from sodium to sodium chloride based on molar mass. This method is practical and cost-effective for large epidemiological studies, although repeated 24-h urine collection remains the gold standard for sodium assessment. Estimates derived from spot urine samples are subject to within-person and between-person variation and may also be influenced by sampling time, renal function, and the applicability of the prediction equation to the target population. These factors may introduce measurement error, lead to misclassification of individual DSI, and attenuate associations because of regression dilution bias. Therefore, the findings should be interpreted primarily at the population level rather than as precise estimates of individual salt intake.

### 2.5 Variable selection strategy

The original dataset included multiple anthropometric and cardiometabolic indicators. To keep the analysis focused and limit false-positive findings from multiple comparisons, we used correlation analysis to guide variable selection. Pearson correlation coefficients were calculated between each cardiometabolic indicator and BMI, WHR, and DSI, with heatmaps used to visualize the overall pattern. Indicators showing relatively strong correlations with both BMI and DSI were prioritized. We also took clinical relevance into account and retained markers reflecting lipid metabolism, urate metabolism, liver function, renal function, and blood pressure.

### 2.6 Statistical analysis

Continuous variables are expressed as mean ± standard deviation (Mean ± SD), categorical variables as frequency (percentage). Between-group comparisons used one-way analysis of variance (ANOVA) for continuous variables and χ^2^ test for categorical variables. Variable correlations were assessed using Pearson correlation coefficients, with |*r*| ≥ 0.3 defined as moderate or higher correlation. Multiple linear regression used the enter method, with BMI or DSI as independent variables, each cardiometabolic indicator as a dependent variable, while controlling for age and sex. Regression associations between BMI and DSI with each indicator were analyzed. Restricted cubic spline (RCS) analysis used 3 knots (at predictor variable 10th, 50th, and 90th percentiles) to assess non-linear dose–response relationships between BMI and DSI, with each cardiometabolic indicator. RCS models were constructed using ordinary least squares (OLS) regression, with analysis of variance (ANOVA) obtaining *p*-values for overall association (P-overall) and non-linearity test (*P*-non-linear). *P*-overall<0.05 indicates an association between predictor and outcome variables; *P*-non-linear<0.05 indicates non-linear characteristics. In total, four models were constructed for each outcome: (1) unadjusted; (2) age-adjusted; (3) sex-adjusted; (4) fully adjusted (controlling for both age and sex). RCS four-panel plots displayed predicted values and 95% confidence intervals for each indicator under different adjustment strategies. Based on fully adjusted model prediction curves, major inflection points were identified through slope change rate calculation.

Moderating effect analysis used hierarchical regression models. Model 1 included BMI, DSI, age, and sex as independent variables; Model 2 added the BMI × DSI interaction term to Model 1. If the interaction term regression coefficient was significant (*p* < 0.05), DSI had a significant moderating effect on the BMI-dependent variable relationship. ΔR^2^ assessed the incremental contribution of the interaction term to the explanatory power of the model. For significant moderating effects, the Johnson–Neyman (J-N) technique determined DSI threshold intervals. J-N method identifies moderator critical values based on 95% confidence interval of conditional effects intersecting the zero line, where above or below this critical value, independent variable conditional effects on dependent variables change from statistically significant to non-significant (or vice versa). J-N moderating effect plots displayed DSI values on the horizontal axis, BMI conditional effects (slopes) on dependent variables on the vertical axis, with shaded areas representing 95% confidence intervals and dashed lines marking J-N threshold positions. Sensitivity analyses stratified participants by age (<60 years as young and middle-aged, ≥60 years as elderly) and sex, repeating moderating effect and J-N analyses in each subgroup to test result robustness and explore effect heterogeneity. J-N moderating effect plots were created separately for the total population and each subgroup for comparison. All statistical analyses used R 4.3.0 software, with main packages including: rms (RCS analysis), interactions (moderating effect and J-N analysis), ggplot2 (data visualization), and corrplot (correlation heatmap). All tests were two-sided, with *p* < 0.05 considered statistically significant, although results close to this threshold were interpreted cautiously.

## 3 Results

### 3.1 Baseline characteristics

The 1,866 participants had an overall mean age of 42.92 ± 16.94 years, a mean body mass index (BMI) of 25.23 ± 4.03 kg/m^2^, and a mean daily salt intake (DSI) of 9.50 ± 2.18 g (Table 1). Significant differences were observed across all four age and sex subgroups (all *p* < 0.01). Young and middle-aged men (*n* = 1,144) presented the highest mean BMI (26.09 ± 4.02 kg/m^2^) and DSI (10.57 ± 1.85 g/d), with salt intake more than double the WHO-recommended limit of 5 g/d. In contrast, elderly women (*n* = 161) recorded the lowest mean DSI (7.01 ± 1.74 g/d) and a mean BMI of 24.34 ± 3.59 kg/m^2^. Systolic blood pressure increased with age, averaging 137.49 ± 17.51 mmHg in elderly men and 133.78 ± 19.08 mmHg in elderly women, compared to 122.15 ± 14.56 mmHg in young and middle-aged men and 110.84 ± 15.60 mmHg in young and middle-aged women.

**Table 1:** Baseline characteristics of study participants stratified by sex and age.

**Characteristic**	**Young male (*n* = 1,144)**	**Elderly male (*n* = 189)**	**Young female (*n* = 372)**	**Elderly female (*n* = 161)**	**Overall** **(*n* = 1,866)**
Demographic and anthropometric					
Age, y	34.75 ± 9.35	71.97 ± 8.65	40.57 ± 9.82	72.13 ± 7.92	42.92 ± 16.94
BMI, kg/m^2^	26.09 ± 4.02	25.32 ± 3.33	22.92 ± 3.53	24.34 ± 3.59	25.23 ± 4.03
WHR	0.87 ± 0.06	0.91 ± 0.06	0.78 ± 0.05	0.86 ± 0.06	0.86 ± 0.07
Dietary intake					
Daily salt intake, g	10.57 ± 1.85	8.89 ± 1.77	7.68 ± 1.62	7.01 ± 1.74	9.50 ± 2.18
Blood pressure					
SBP, mm Hg	122.15 ± 14.56	137.49 ± 17.51	110.84 ± 15.60	133.78 ± 19.08	122.45 ± 17.36
DBP, mm Hg	74.47 ± 10.81	72.71 ± 11.01	69.76 ± 10.38	67.91 ± 11.22	72.79 ± 11.03
Glucose					
GLU, mmol/L	5.63 ± 1.33	6.69 ± 1.94	5.33 ± 0.61	6.41 ± 1.79	5.74 ± 1.41
Lipid profile					
TC, mmol/L	4.90 ± 0.92	5.02 ± 1.15	4.89 ± 0.83	5.26 ± 1.20	4.94 ± 0.96
TG, mmol/L	1.65 ± 1.32	1.49 ± 0.86	1.07 ± 0.68	1.40 ± 0.61	1.50 ± 1.15
HDL-C, mmol/L	1.11 ± 0.24	1.22 ± 0.28	1.40 ± 0.28	1.36 ± 0.34	1.20 ± 0.29
LDL-C, mmol/L	2.79 ± 0.69	2.68 ± 0.84	2.62 ± 0.64	2.74 ± 0.90	2.74 ± 0.72
sdLDL, mmol/L	0.91 ± 0.25	0.88 ± 0.29	0.82 ± 0.22	0.89 ± 0.29	0.89 ± 0.26
Lpa, mg/L	155.03 ± 212.64	207.94 ± 238.67	196.75 ± 241.79	330.91 ± 335.30	183.87 ± 239.14
Apolipoprotein					
ApoA1, g/L	1.36 ± 0.17	1.46 ± 0.19	1.51 ± 0.19	1.57 ± 0.21	1.42 ± 0.19
ApoB, g/L	0.94 ± 0.21	0.94 ± 0.26	0.86 ± 0.20	0.95 ± 0.25	
ApoB/ApoA1	0.70 ± 0.17	0.65 ± 0.20	0.58 ± 0.15	0.61 ± 0.18	0.662 ± 0.180
Liver function					
AST, U/L	22.78 ± 13.56	21.31 ± 6.33	18.81 ± 6.51	21.27 ± 8.04	
ALT, U/L	34.10 ± 30.50	20.37 ± 10.37	18.27 ± 14.49	19.16 ± 15.74	28.27 ± 26.43
*γ*-GT, U/L	34.93 ± 31.76	32.85 ± 65.88	19.16 ± 13.66	19.61 ± 9.39	
Renal function					
Cr, μmol/L	77.23 ± 11.13	81.62 ± 17.53	55.74 ± 9.44	62.22 ± 13.02	72.10 ± 15.04
ACR, mg/g	14.31 ± 82.29	65.87 ± 278.01	10.73 ± 15.09	27.00 ± 93.03	
Other biomarkers					
UA, μmol/L	398.10 ± 81.29	358.32 ± 77.18	272.52 ± 63.16	296.06 ± 65.20	360.25 ± 92.62

Data are presented as mean ± SD unless otherwise indicated. Young participants were defined as age <60 years; elderly participants were defined as age ≥60 years. *p*-values were calculated using the Kruskal–Wallis test for comparison among the 4 subgroups, and all were statistically significant (*p* < 0.001).

Metabolic profiles showed clear sex disparities. Women exhibited higher levels of HDL-C and apolipoprotein A1 (ApoA1), while men had substantially higher serum uric acid (UA). Among young and middle-aged men, UA reached 398.10 ± 81.29 μmol/L, approaching the diagnostic threshold for hyperuricemia, and their ApoB/ApoA1 ratio was the least favorable (0.698 ± 0.174). Conversely, young and middle-aged women had the lowest UA (272.52 ± 63.16 μmol/L) and the most favorable ApoB/ApoA1 ratio (0.576 ± 0.153). Serum creatinine was consistently higher in men, aligning with known sex-related differences in muscle mass. Alanine aminotransferase (ALT) was markedly elevated in young and middle-aged men (34.10 ± 30.50 U/L) relative to the other three subgroups, whose ALT levels were similar and ranged from 18.27 to 20.37 U/L.

### 3.2 Variable selection and correlation analysis

**Figure 1:**
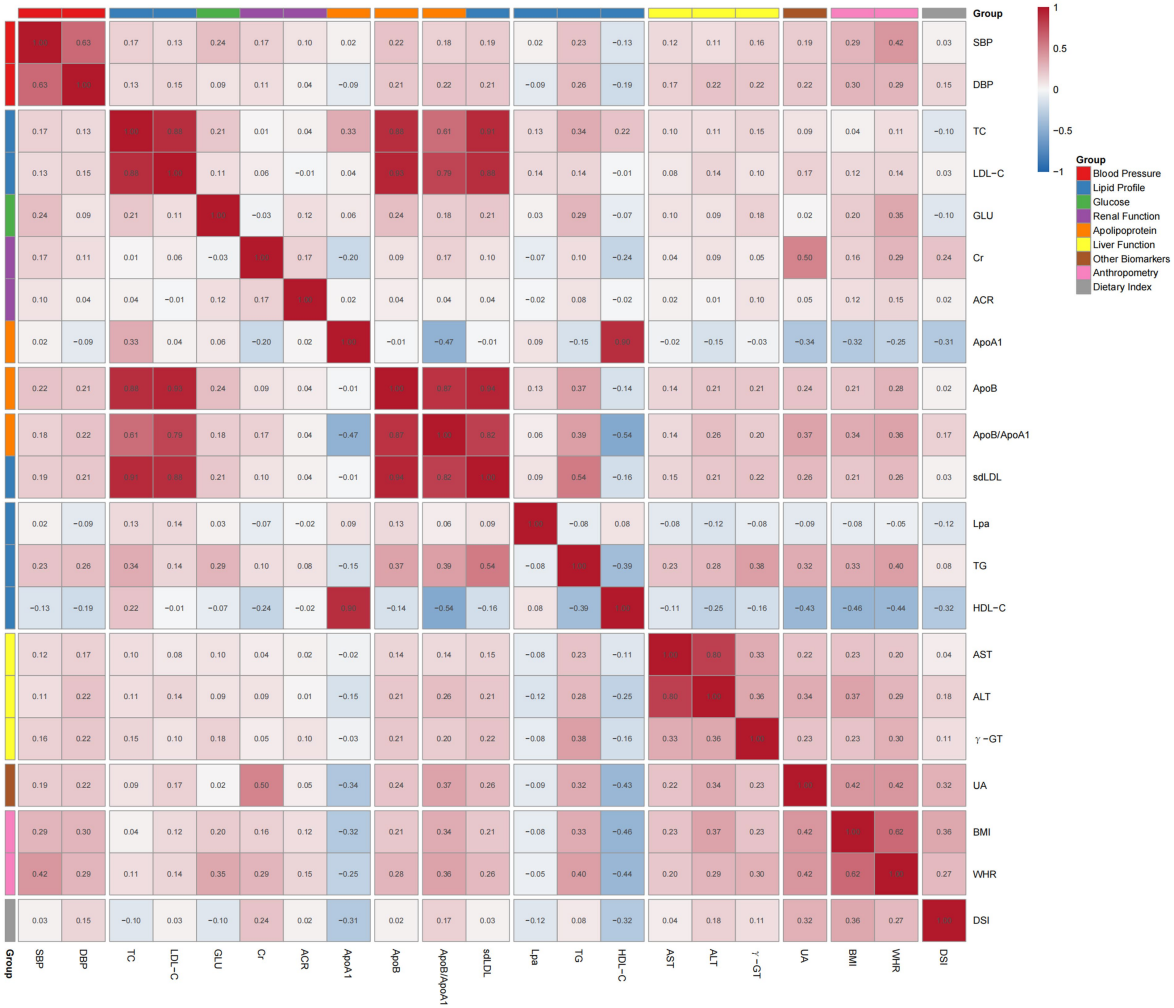
Correlation heatmap between variables.

Figure 1 displays correlations between all variables. BMI correlated strongly with WHR (*r* = 0.62, *p* < 0.001) and moderately with DSI (*r* = 0.36, *p* < 0.001). BMI showed moderate negative correlations with HDL-C (*r* = −0.46) and ApoA1 (*r* = −0.32), and moderate positive correlations with UA (*r* = 0.42), ALT (*r* = 0.37), ApoB/ApoA1 (*r* = 0.34), TG (*r* = 0.33), DBP (*r* = 0.30), and SBP (*r* = 0.30). Table 2 further lists detailed correlation coefficients. Among absolute correlations with BMI, WHR ranked first (*r* = 0.62), followed by HDL-C (*r* = −0.46), UA (*r* = 0.42), and ALT (*r* = 0.37). Similarly, the top 10 variables correlated with WHR and DSI were analyzed separately. DSI-BMI correlation coefficient (0.36) exceeded DSI-WHR correlation (0.27), thus BMI was selected as the independent variable. Synthesizing three analyses, HDL-C, UA, ApoA1, Cr, ALT, ApoB/ApoA1, and DBP, showing stronger DSI correlations, were selected as cardiometabolic outcome indicators for subsequent analyses.

**Table 2:** Top 10 variables most correlated with BMI, WHR, and DSI.

**Rank**	**BMI-correlated variables**	**r**	**WHR-correlated variables**	**r**	**DSI-correlated variables**	**r**
1	WHR	0.62	BMI	0.62	BMI	0.36
2	HDL-C	−0.46	HDL-C	−0.44	HDL-C	−0.32
3	UA	0.42	SBP	0.42	UA	0.32
4	ALT	0.37	UA	0.42	ApoA1	−0.31
5	DSI	0.36	TG	0.40	WHR	0.27
6	ApoB/ApoA1	0.34	ApoB/ApoA1	0.36	Cr	0.24
7	TG	0.33	GLU	0.35	ALT	0.18
8	ApoA1	−0.32	γ-GT	0.30	ApoB/ApoA1	0.17
9	DBP	0.30	Cr	0.30	DBP	0.15
10	SBP	0.29	ALT	0.29	Lpa	−0.12

r = Pearson correlation coefficient; all correlation coefficients. *p* < 0.001.

### 3.3 Regression analysis of BMI and DSI with cardiometabolic indicators

Tables 3, 4 and Supplementary material 1 present regression analysis results of BMI and DSI with 7 cardiometabolic indicators, including total population, sex stratification, age stratification, and analyses adjusted for sex and age. In the total population, BMI was associated significantly and negatively with HDL-C (*β* = −0.033, *p* < 0.001), with the association weakening to *β* = −0.027 (p < 0.001) after controlling for age and sex. Sex stratification revealed HDL-C decreased 0.025 mmol/L per unit BMI increase in men (*p* < 0.001) and 0.032 mmol/L in women (*p* < 0.001). Age stratification showed no obvious differences, with regression coefficients of −0.033 for both young and middle-aged and elderly populations (both *p* < 0.001). BMI-UA association showed age-dependent characteristics. In unadjusted models, each 1 kg/m^2^ BMI increase was associated with 9.670 μmol/L UA increase (*p* < 0.001); after controlling for confounding factors, the effect weakened to 6.526 μmol/L (*p* < 0.001), a 33% reduction. Men showed greater UA increase (*β* = 6.874) than women (*β* = 5.354). Effect size in the young and middle-aged population (*β* = 10.351) markedly exceeded that of the elderly (*β* = 4.493), suggesting age moderates BMI-UA association. The BMI-ALT association also showed sex differences. In adjusted models, each 1 kg/m^2^ BMI increase is associated with 2.166 U/L ALT increase (*p* < 0.001). Sex stratification revealed that the effect size in men (*β* = 2.614) significantly exceeded that of women (*β* = 0.773), reflecting sex differences in fatty liver susceptibility. BMI associations with ApoA1, ApoB/ApoA1, and DBP remained stable before and after adjustment. ApoA1 decreased with BMI increase (adjusted *β* = −0.011, *p* < 0.001), ApoB/ApoA1 ratio increased (*β* = 0.013, *p* < 0.001), and DBP increased (*β* = 0.714, *p* < 0.001). BMI showed weak positive correlation with Cr in unadjusted models (*β* = 0.616, *p* < 0.001), but the association disappeared after controlling for age and sex (*β* = −0.065, *p* = 0.357), indicating age and sex were the main drivers of the BMI-Cr association.

**Table 3:** Stratified linear regression analysis of BMI and DSI with cardiometabolic parameters.

Outcome	Predictor	Overall population (*N* = 1866)	Male (*N* = 1,333)	Female (*N* = 533)	Age <60 years (*N* = 1,517)	Age ≥60 years (*N* = 350)
		*β* (SE), P, *R*^2^				
HDL-C, mmol/L	BMI	−0.033 (0.001)	−0.025 (0.002)	−0.032 (0.003)	−0.033 (0.002)	−0.033 (0.005)
		*P* < 0.001, *R*^2^ = 0.210	*P* < 0.001, *R*^2^ = 0.155	*P* < 0.001, *R*^2^ = 0.149	*P* < 0.001, *R*^2^ = 0.231	*P* < 0.001, *R*^2^ = 0.134
	DSI	−0.041 (0.003)	−0.012 (0.004)	−0.030 (0.008)	−0.041 (0.003)	−0.034 (0.008)
		*P* < 0.001, *R*^2^ = 0.101	*P* < 0.001, *R*^2^ = 0.009	*P* < 0.001, *R*^2^ = 0.027	*P* < 0.001, *R*^2^ = 0.098	*P* < 0.001, *R*^2^ = 0.045
UA, μmol/L	BMI	9.670 (0.484)	6.874 (0.537)	5.354 (0.743)	10.351 (0.521)	4.493 (1.181)
		*P* < 0.001, *R*^2^ = 0.177	*P* < 0.001, *R*^2^ = 0.109	*P* < 0.001, *R*^2^ = 0.089	*P* < 0.001, *R*^2^ = 0.206	*P* < 0.001, *R*^2^ = 0.040
	DSI	12.896 (0.899)	0.814 (1.164)	−2.284 (1.662)	13.022 (1.058)	6.966 (2.074)
		*P* < 0.001, *R*^2^ = 0.099	P = 0.485, R^2^ < 0.001	P = 0.170, *R*^2^ = 0.004	*P* < 0.001, *R*^2^ = 0.091	*P* < 0.001, *R*^2^ = 0.031
ApoA1, g/L	BMI	−0.015 (0.001)	−0.010 (0.001)	−0.013 (0.002)	−0.014 (0.001)	−0.018 (0.003)
		*P* < 0.001, *R*^2^ = 0.100	*P* < 0.001, *R*^2^ = 0.056	*P* < 0.001, *R*^2^ = 0.055	*P* < 0.001, *R*^2^ = 0.105	*P* < 0.001, *R*^2^ = 0.087
	DSI	−0.027 (0.002)	−0.012 (0.002)	−0.020 (0.005)	−0.023 (0.002)	−0.021 (0.006)
		*P* < 0.001, *R*^2^ = 0.099	*P* < 0.001, *R*^2^ = 0.017	*P* < 0.001, *R*^2^ = 0.029	*P* < 0.001, *R*^2^ = 0.076	*P* < 0.001, *R*^2^ = 0.040
Cr, μmol/L	BMI	0.616 (0.085)	−0.073 (0.086)	−0.011 (0.133)	0.646 (0.086)	0.462 (0.282)
		*P* < 0.001, *R*^2^ = 0.027	*p* = 0.397, *R*^2^ = 0.001	*p* = 0.935, *R*^2^ < 0.001	*P* < 0.001, *R*^2^ = 0.036	*p* = 0.102, *R*^2^ = 0.008
	DSI	1.590 (0.149)	−0.908 (0.174)	−1.477 (0.277)	1.893 (0.160)	1.380 (0.489)
		*P* < 0.001, *R*^2^ = 0.057	*P* < 0.001, *R*^2^ = 0.020	*P* < 0.001, *R*^2^ = 0.051	*P* < 0.001, *R*^2^ = 0.085	*p* = 0.005, *R*^2^ = 0.022
ALT, U/L	BMI	2.417 (0.141)	2.614 (0.188)	0.773 (0.176)	2.665 (0.162)	0.616 (0.199)
		*P* < 0.001, *R*^2^ = 0.136	*P* < 0.001, *R*^2^ = 0.127	*P* < 0.001, *R*^2^ = 0.035	*P* < 0.001, *R*^2^ = 0.152	*p* = 0.002, *R*^2^ = 0.027
	DSI	2.093 (0.266)	0.977 (0.411)	0.247 (0.383)	1.939 (0.329)	0.419 (0.352)
		*P* < 0.001, *R*^2^ = 0.032	*p* = 0.017, *R*^2^ = 0.004	*p* = 0.520, *R*^2^ = 0.001	*P* < 0.001, *R*^2^ = 0.022	*p* = 0.235, *R*^2^ = 0.004
ApoB/ApoA1 ratio	BMI	0.015 (0.001)	0.013 (0.001)	0.010 (0.002)	0.017 (0.001)	0.003 (0.003)
		*P* < 0.001, *R*^2^ = 0.113	*P* < 0.001, *R*^2^ = 0.089	*P* < 0.001, *R*^2^ = 0.053	*P* < 0.001, *R*^2^ = 0.157	*p* = 0.365, *R*^2^ = 0.002
	DSI	0.013 (0.002)	0.002 (0.003)	0.001 (0.004)	0.014 (0.002)	0.005 (0.005)
		*P* < 0.001, *R*^2^ = 0.028	*p* = 0.388, *R*^2^ = 0.001	*p* = 0.741, R^2^ < 0.001	*P* < 0.001, *R*^2^ = 0.031	*p* = 0.311, *R*^2^ = 0.003
DBP, mmHg	BMI	0.820 (0.061)	0.714 (0.073)	0.721 (0.125)	0.869 (0.064)	0.447 (0.173)
		*P* < 0.001, *R*^2^ = 0.089	*P* < 0.001, *R*^2^ = 0.067	*P* < 0.001, *R*^2^ = 0.059	*P* < 0.001, *R*^2^ = 0.109	*p* = 0.010, *R*^2^ = 0.019
	DSI	0.719 (0.112)	0.171 (0.154)	0.372 (0.274)	0.481 (0.128)	1.397 (0.296)
		*P* < 0.001, *R*^2^ = 0.022	*p* = 0.268, *R*^2^ = 0.001	*p* = 0.175, *R*^2^ = 0.003	*P* < 0.001, *R*^2^ = 0.009	*P* < 0.001, *R*^2^ = 0.060

Data are presented as regression coefficient (standard error), *P*-value, and coefficient of determination (*R*^2^). All models were unadjusted for covariates.

**Table 4:** Comparison of regression coefficients before and after adjustment for age and sex.

**Outcome**	**Predictor**	**Unadjusted model**	**Adjusted model†**	**Change in effect**	***P*-value change**	**Change in model fit**
		*β* (95% CI)	*β* (95% CI)	Δβ (%)		Δ*R*^2^ (%)
HDL-C, mmol/L	BMI	−0.033 (−0.035, −0.031)	−0.027 (−0.029, −0.025)	−18.2	<0.001 → <0.001	0.210 → 0.296 (+41.0)
	DSI	−0.041 (−0.047, −0.035)	−0.014 (−0.022, −0.006)	−65.9	<0.001 → <0.001	0.101 → 0.177 (+75.2)
UA, μmol/L	BMI	9.670 (8.721, 10.619)	6.526 (5.667, 7.385)	−32.5	<0.001 → <0.001	0.177 → 0.385 (+117.5)
	DSI	12.896 (11.134, 14.658)	−1.690 (−3.683, 0.303)	Direction reversed	<0.001 → 0.097	0.099 → 0.313 (+216.2)
ApoA1, g/L	BMI	−0.015 (−0.017, −0.013)	−0.011 (−0.013, −0.009)	−26.7	<0.001 → <0.001	0.100 → 0.233 (+133.0)
	DSI	−0.027 (−0.031, −0.023)	−0.006 (−0.010, −0.002)	−77.8	<0.001 → 0.007	0.099 → 0.187 (+88.9)
Cr, μmol/L	BMI	0.616 (0.449, 0.783)	−0.065 (−0.204, 0.074)	Direction reversed	<0.001 → 0.357	0.027 → 0.389 (+1340.7)
	DSI	1.590 (1.298, 1.882)	−0.710 (−1.014, −0.406)	Direction reversed	<0.001 → <0.001	0.057 → 0.396 (+594.7)
ALT, U/L	BMI	2.417 (2.141, 2.693)	2.166 (1.882, 2.450)	−10.4	<0.001 → <0.001	0.136 → 0.169 (+24.3)
	DSI	2.093 (1.571, 2.615)	0.231 (−0.434, 0.896)	−89.0	<0.001 → 0.494	0.032 → 0.070 (+118.8)
ApoB/ApoA1 ratio	BMI	0.015 (0.013, 0.017)	0.013 (0.011, 0.015)	−13.3	<0.001 → <0.001	0.113 → 0.143 (+26.5)
	DSI	0.013 (0.009, 0.017)	0.003 (−0.001, 0.007)	−76.9	<0.001 → 0.168	0.028 → 0.071 (+153.6)
DBP, mmHg	BMI	0.820 (0.700, 0.940)	0.714 (0.591, 0.837)	−12.9	<0.001 → <0.001	0.089 → 0.105 (+18.0)
	DSI	0.719 (0.499, 0.939)	0.319 (0.039, 0.599)	−55.6	<0.001 → 0.026	0.022 → 0.046 (+109.1)

†Adjusted for age (continuous) and sex (binary) using multiple linear regression; *N* = 1866 for all models. Δβ (%) = [(Adjusted β - Unadjusted β) / Unadjusted β] × 100%. Bold text indicates >50% reduction in effect size or reversal of association direction.

Daily salt intake associations with most metabolic indicators substantially weakened or disappeared after adjusting for age and sex. This pattern suggests that the apparent effect of DSI was more sensitive to confounding than that of BMI. Unadjusted models showed DSI negatively correlated with HDL-C (*β* = −0.041, *p* < 0.001), but after adjustment, the effect size decreased 66% (*β* = −0.014, *p* < 0.001). DSI–HDL-C association in women (*β* = −0.030) exceeded that of men (*β* = −0.012). In DSI–UA association, unadjusted models showed significant positive correlation (*β* = 12.896, *p* < 0.001), but after controlling for age and sex, association reversed and lost significance (*β* = −1.690, *p* = 0.097); no significant associations appeared in men (*p* = 0.485) or women (*p* = 0.170); in young and middle-aged population, unadjusted *β* = 13.022 (*p* < 0.001), but this association primarily reflected age confounding. Similar patterns appeared in the DSI-Cr association. Unadjusted models showed positive correlation (*β* = 1.590, *p* < 0.001), but after adjustment, reversed to negative correlation (*β* = −0.710, p < 0.001); both men and women showed negative correlations, with women showing stronger effects (*β* = −1.477 vs. − 0.908). DSI associations with ALT and ApoB/ApoA1 completely disappeared after controlling for confounding factors. ALT regression coefficient dropped from 2.093 (*p* < 0.001) to 0.231 (*p* = 0.494), ApoB/ApoA1 from 0.013 (*p* < 0.001) to 0.003 (*p* = 0.168). Only DBP maintained a weak correlation, with adjusted *β* = 0.319 (*p* = 0.026), but the effect size decreased 56%. BMI effects on metabolic indicators remained relatively stable after adjustment, with effect sizes for HDL-C, UA, and ApoA1 decreasing only 19, 33, and 27%, respectively. DSI effects proved highly sensitive to age and sex, with effect sizes for HDL-C and ApoA1 sharply decreasing 66 and 77%, respectively, and even association direction reversal for UA and Cr. Model goodness-of-fit (*R*^2^) generally improved after including age and sex, such as HDL-C model *R*^2^ increasing from 0.210 to 0.296 and UA model from 0.177 to 0.385.

### 3.4 Non-linear relationships of BMI and DSI with cardiometabolic indicators

Restricted cubic spline (RCS) analysis placed knots at BMI 10th, 50th, and 90th percentiles (20.32, 24.80, 30.42 kg/m^2^) and corresponding DSI percentiles (6.67, 9.56, and 12.28 g/d) (Supplementary material 2 and Tables 5, 6). As shown in Table 5 and Supplementary material 2, after controlling for age and sex, BMI showed significant non-linear relationships with multiple cardiometabolic indicators. Based on slope change rate analysis of fitted curves, major inflection points concentrated consistently at BMI 24.95 kg/m^2^, corresponding to normal weight to overweight transition (WHO Asian population standard 25 kg/m^2^). Among lipoprotein metabolism indicators, HDL-C showed a significant non-linear negative correlation with BMI (*P*-overall<0.001, *P*-non-linear < 0.001, *R*^2^ = 0.319), with curves showing HDL-C rapidly declining as BMI increased. At the inflection point (24.95 kg/m^2^, 1.12 mmol/L), the slope was −0.033 mmol/L per kg/m^2^, after which the decline rate markedly slowed. Average slope in the interval from knot 1 to knot 2 (20.32–24.80 kg/m^2^) was −0.047 mmol/L per kg/m^2^, slowing to −0.018 mmol/L per kg/m^2^ in the interval from knot 2 to knot 3 (24.80–30.42 kg/m^2^), a 62% reduction, suggesting HDL-C sensitivity to BMI changes peaks in normal weight range. ApoA1 exhibited a highly similar non-linear pattern (*P*-overall<0.001, *P*-non-linear<0.001, *R*^2^ = 0.253), with inflection point also at 24.95 kg/m^2^ (1.36 g/L, slope −0.015 g/L per kg/m^2^), interval slopes slowing from −0.025 g/L per kg/m^2^ to −0.005 g/L per kg/m^2^, a 79% reduction. ApoB/ApoA1 ratio showed significant non-linear positive correlation with BMI (*P*-overall<0.001, *P*-non-linear<0.001, *R*^2^ = 0.157), inflection point at 24.95 kg/m^2^ (0.70, slope 0.016), interval slopes slowing from 0.018 to 0.007 (61% reduction). Consistent inflection point around 25 kg/m^2^ for these three lipoprotein indicators suggests that the normal weight to overweight transition stage represents a critical physiological window where lipid metabolism balance undergoes a key transformation.

**Table 5:** Restricted cubic spline analysis of BMI and cardiovascular metabolic indicators (fully adjusted model).

Indicator	*P*-overall	*P*-non-linear	*R* ^2^	Knot 1ᵃ	Knot 2ᵃ	Knot 3ᵃ	Inflection pointᵇ	Slope interval 1ᶜ	Slope interval 2ᵈ	Slope change
HDL-C (mmol/L)	<0.001***	<0.001***	0.319	(20.32, 1.33)	(24.80, 1.12)	(30.42, 1.02)	(24.95, 1.12)	−0.047	−0.018	−62%
ApoA1 (g/L)	<0.001***	<0.001***	0.253	(20.32, 1.47)	(24.80, 1.36)	(30.42, 1.33)	(24.95, 1.36)	−0.025	−0.005	−79%
ApoB/ ApoA1	<0.001***	<0.001***	0.157	(20.32, 0.85)	(24.80, 0.93)	(30.42, 0.97)	(24.95, 0.70)	0.018	0.007	−61%
UA (μmol/L)	<0.001***	0.011*	0.387	(20.32, 351)	(24.80, 390)	(30.42, 421)	(24.95, 390.3)	8.70	5.52	−37%
ALT (U/L)	<0.001***	0.054	0.171	(20.32, 21.6)	(24.80, 29.3)	(30.42, 42.7)	(24.95, 29.48)	1.72	2.38	+38%
DBP (mmHg)	<0.001***	0.068	0.107	(20.32, 70.7)	(24.80, 73.0)	(30.42, 77.6)	(24.95, 73.09)	0.51	0.82	+61%
Cr (μmol/L)	0.352	0.266	0.390	(20.32, 77.6)	(24.80, 77.9)	(30.42, 77.2)	–	0.07	−0.12	-

*P < 0.05, **P < 0.01, ***P < 0.001. All analyses were adjusted for age and sex. ᵃ Knot coordinates in format (BMI kg/m^2^, predicted value), placed at 10th, 50th, and 90th percentiles of BMI. ᵇ Inflection point calculated based on slope change rate from fully adjusted model curves, format (BMI kg/m^2^, predicted value). ᶜ Slope Interval 1: average rate of change between Knot 1 and Knot 2. ᵈ Slope Interval 2: average rate of change between Knot 2 and Knot 3. Negative slope change indicates deceleration, positive indicates acceleration. For Cr, the inflection point and slope change are marked as “–” due to a non-significant overall association.

**Table 6:** Restricted cubic spline analysis of DSI and cardiovascular metabolic indicators (fully adjusted model).

**Indicator**	**P-overall**	**P-non-linear**	**R** ^**2**^	**Knot 2ᵃ**	**Inflection Pointᵇ**	**Association Pattern**
DBP (mmHg)	0.002**	0.008**	0.049	(9.56, 74.4)	(9.61, 74.36)	Significant non-linear
HDL-C (mmol/L)	<0.001***	0.121	0.178	(9.56, 1.13)	(9.61, 1.13)	Significant but near-linear
Cr (μmol/L)	<0.001***	0.092	0.397	(9.56, 77.8)	(9.61, 77.84)	Significant but near-linear
ApoA1 (g/L)	0.024*	0.597	0.187	(9.56, 1.37)	(9.61, 1.37)	Weak linear association
UA (μmol/L)	0.249	0.870	0.313	(9.56, 395)	–	Not significant
ApoB/ApoA1	0.286	0.438	0.071	(9.56, 0.69)	–	Not significant
ALT (U/L)	0.786	0.906	0.070	(9.56, 32.4)	–	Not significant

*P < 0.05, **P < 0.01, ***P < 0.001. All analyses were adjusted for age and sex. ᵃ Knot 2 coordinate represents predicted value at DSI 50th percentile (median 9.56 g/d); complete knots at 6.67, 9.56, 12.28 g/d. ᵇ Inflection point calculated from fully adjusted model curves, format (DSI g/d, predicted value). Association pattern classification: Significant non-linear (*P*-overall<0.05 and *P*-non-linear<0.05); Significant but near-linear (*P*-overall<0.05 but *P*-non-linear≥0.05); Weak linear association (*P*-overall 0.01–0.05 with non-significant *P*-non-linear); Not significant (*P*-overall≥0.05). Inflection points not reported for non-significant indicators (marked “–”).

Regarding metabolism-related indicators and blood pressure, UA showed significant non-linear positive correlation with BMI (*P*-overall < 0.001, *P*-non-linear = 0.011, *R*^2^ = 0.387), inflection point at 24.95 kg/m^2^ (390.30 μmol/L, slope 7.11 μmol/L per kg/m^2^). Interval slopes slowed from 8.70 μmol/L per kg/m^2^ to 5.52 μmol/L per kg/m^2^, a 37% reduction, indicating uric acid levels rise most rapidly during the normal weight to overweight transition, subsequently slowing but maintaining a significant upward trend. ALT showed positive correlation with BMI (*P*-overall < 0.001, *R*^2^ = 0.171), with a non-linearity test at borderline significance (*P*-non-linear = 0.054), inflection point at 24.95 kg/m^2^ (29.48 U/L, slope 2.02 U/L per kg/m^2^). ALT interval slopes accelerated from 1.72 U/L per kg/m^2^ to 2.38 U/L per kg/m^2^ (38% increase), contrary to the deceleration pattern of other indicators, suggesting liver enzyme elevation accelerates in overweight populations, possibly reflecting a non-linear increase in fatty liver risk. DBP showed significant positive correlation with BMI (*P*-overall < 0.001, *R*^2^ = 0.107), but non-linearity test did not reach significance (*P*-non-linear = 0.068), inflection point at 24.95 kg/m^2^ (73.09 mmHg, slope 0.65 mmHg per kg/m^2^), interval slopes accelerating from 0.51 mmHg per kg/m^2^ to 0.82 mmHg per kg/m^2^ (61% increase), indicating relationship closer to linear but with slight acceleration at higher BMI ranges. Cr showed no significant association with BMI after adjusting for age and sex (*P*-overall = 0.352, *P*-non-linear = 0.266), suggesting BMI–creatinine association primarily reflects age and sex confounding.

Compared to BMI, DSI relationships with cardiometabolic indicators substantially weakened or disappeared after adjusting for age and sex (Supplementary material 2 and Table 6). DBP was the only indicator maintaining significant non-linearity after adjustment (*P*-overall = 0.002, *P*-non-linear = 0.008, *R*^2^ = 0.049), with an inflection point at 9.61 g/d (74.36 mmHg, slope 0.41 mmHg per g/d). Curves showed DSI had minimal impact on diastolic pressure in the lower intake range (6.67–9.56 g/d) but showed an upward trend at higher intake levels (>9.61 g/d), indicating high salt intake effects on blood pressure involve dose-dependent thresholds. HDL-C showed significant non-linear negative correlation with DSI in unadjusted models (*P*-non-linear<0.001), but after adjusting for age and sex, although overall association remained significant (*P*-overall<0.001), non-linear characteristics markedly weakened (*P*-non-linear = 0.121, *R*^2^ = 0.178), with an inflection point at 9.61 g/d (1.13 mmol/L, slope −0.016 mmol/L per g/d). Cr remained significantly associated with DSI after adjustment (*P*-overall<0.001, *R*^2^ = 0.397), but non-linearity was not significant (*P*-non-linear = 0.092), with an inflection point at 9.61 g/d (77.84 μmol/L, slope −0.77 μmol/L per g/d). ApoA1 showed a weak association with DSI (*P*-overall = 0.024, *R*^2^ = 0.187), but non-linearity was not significant (*P*-non-linear = 0.597). Other indicators (UA, ALT, and ApoB/ApoA1) did not reach significance in DSI associations after adjustment (*P*-overall>0.05), indicating these apparent associations mainly reflected age and sex confounding. For example, UA P-overall decreased from <0.001 unadjusted to 0.249, ApoB/ApoA1 from <0.001 to 0.286. Overall, when BMI served as a predictor, inflection points of all the significant indicators concentrated at 24.95 kg/m^2^, demonstrating significant biological consistency. While the DSI inflection point was 9.61 g/d (exceeding the Chinese Dietary Guidelines’ recommended upper limit of 6 g/d), only DBP maintained a robust non-linear association, with other indicators’ associations essentially disappearing after confounding factor adjustment, suggesting DSI effects on cardiometabolic indicators are weaker than BMI.

### 3.5 Moderating effects of DSI on BMI–cardiometabolic indicator relationships and sensitivity analysis

**Figure 2:**
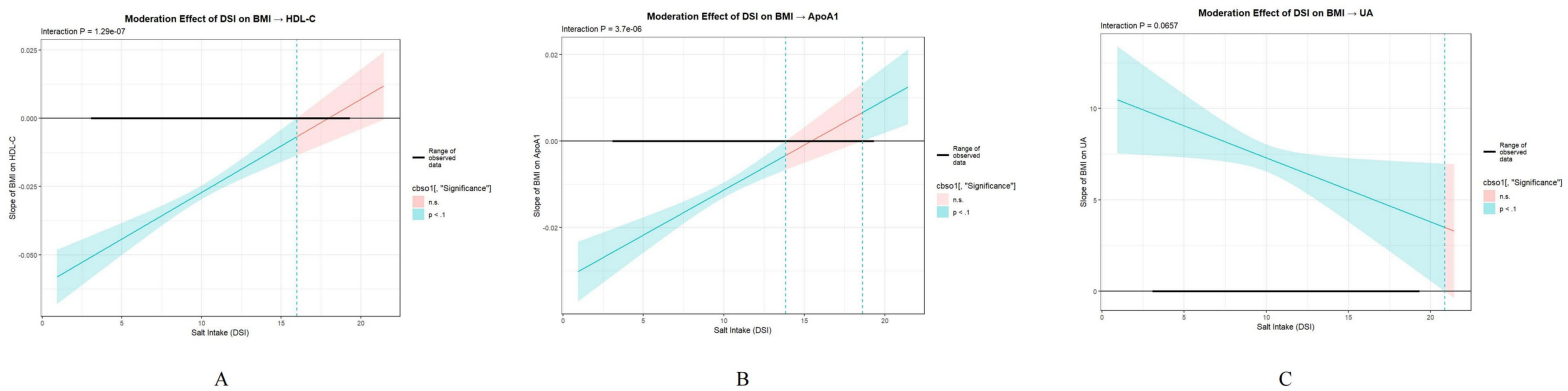
Johnson–Neyman moderation effect analysis in the total population. **(A)** HDL-C; **(B)** ApoA1; **(C)** UA. The horizontal axis represents DSI values (g/d), and the vertical axis represents the conditional effect (slope) of BMI on the dependent variable. The solid fitted line represents the estimated conditional effect, the shaded area represents the 95% confidence interval, the horizontal black solid line represents the data range, and the vertical dashed lines mark the J-N threshold positions. The intersection of the confidence interval with the zero line indicates the threshold.

Hierarchical regression analysis showed DSI exerts selective moderating effects on BMI-cardiometabolic indicator relationships (Table 7). After controlling for age and sex, DSI showed significant moderating effects on the relationships of BMI with HDL-C and ApoA1, whereas the interaction for UA was suggestive but did not reach the conventional threshold for statistical significance. For HDL-C, the interaction term BMI × DSI regression coefficient was 0.0034 (SE = 0.0006, *p* < 0.001), model *R*^2^ = 0.307. Positive interaction effect indicated that as DSI increased, the negative effect of BMI on HDL-C somewhat weakened. Johnson–Neyman analysis identified two critical DSI thresholds: lower limit 15.69 g and upper limit 22.68 g (Figure 2A). When DSI < 15.69 g, the conditional effect of BMI on HDL-C was significantly negative, with 95% CI excluding zero; when DSI ranged 15.69–22.68 g, the confidence interval of the effect included zero and lost statistical significance; when DSI > 22.68 g, the conditional effect might regain significance. In the sample, 99.79% of participants had DSI < 15.69 g, with only 1 case exceeding 22.68 g. ApoA1 showed a highly similar moderating pattern, interaction coefficient 0.0021 (SE = 0.0004, *p* < 0.001), model *R*^2^ = 0.242, DSI thresholds at lower limit 13.58 g and upper limit 19.40 g (Figure 2B), with 97.59% of participants having DSI < 13.58 g and only 1 case exceeding 19.40 g. For UA, the interaction term was in the opposite direction to that observed for HDL-C and ApoA1, but it did not reach the conventional threshold for statistical significance (*β* = −0.377, SE = 0.190, *p* = 0.066). This pattern was therefore interpreted cautiously. Although the *p*-value was close to 0.05, the result was treated as suggestive rather than statistically significant. Negative interaction term indicated DSI increase weakened the positive effect of BMI on UA, with DSI moderating a lower threshold at 19.51 g (Figure 2C), but again with only one participant exceeding this threshold.

**Table 7:** Moderation effect analysis of DSI on the relationship between BMI and cardiometabolic indicators.

**Dependent variable**	***R*** ^**2**^	***β* interaction (SE)**	***t-*value**	***P*-value**	**Moderation effect**	**J-N threshold (g/d)**	**Sample distribution**
HDL-C	0.307	0.0034(0.0006)	5.34	<0.001	Significant	15.97	99.89% < lower limit
ApoA1	0.242	0.0021(0.0004)	4.71	<0.001	Significant	13.84, 18.62	98.07% < lower limit
UA	0.398	−0.377(0.190)	−1.99	0.066	Suggestive, not statistically significant	20.86, −	100.00% < lower limit
ApoB/ApoA1	0.073	0.0003(0.0006)	0.62	0.537	Non-significant	–	–
Cr	0.396	−0.033(0.031)	−1.08	0.278	Non-significant	–	–
ALT	0.174	0.066(0.063)	1.04	0.301	Non-significant	–	–
DBP	0.106	0.005(0.028)	0.19	0.846	Non-significant	–	–

Models included BMI, DSI, age, sex, and the BMI×DSI interaction term; βinteraction indicates the regression coefficient of the interaction term; sample distribution refers to the proportion of the population below the lower threshold; statistical analysis was performed using the Hayes–Matthes computational procedure (23). *P*-values close to 0.05 were interpreted cautiously, together with the overall pattern of results.

**Figure 3:**
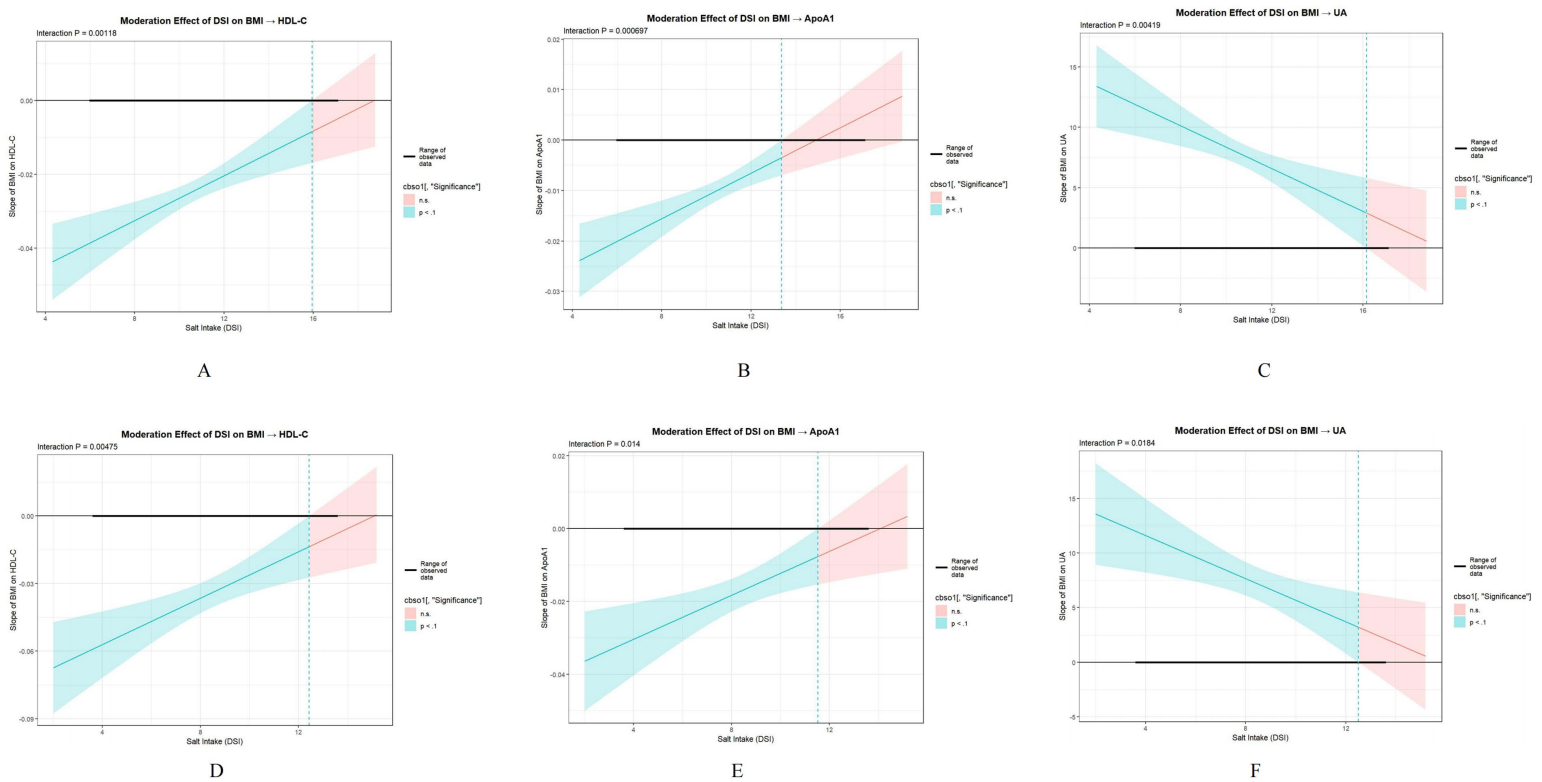
Johnson–Neyman moderation effect analysis stratified by age and sex. **(A–C)** Young and middle-aged men; **(D–F)** young and middle-aged women. Legend same as Figure 2.

Sensitivity analysis showed significant age heterogeneity and sex differences in moderating effects. In the young and middle-aged population (<60 years), DSI moderating effects remained highly significant, demonstrating sex-specific threshold patterns; whereas in the elderly population (≥60 years), all interaction terms failed to reach statistical significance, with DSI moderating effects absent. Among young and middle-aged men (*n* = 1,144), the interaction effects for all three indicators were highly significant. The lower DSI moderating threshold for HDL-C was 15.95 g (*p* = 0.0012), with 99.91% of this subgroup having an intake below this value. Uric acid (UA) exhibited a threshold of 16.15 g (*p* = 0.0042), while the threshold for ApoA1 was 13.38 g (*p* = 0.0007), with 98.03% of young and middle-aged men falling below this level. Figures 3A–C shows that when DSI was below these thresholds, BMI associations with each indicator were significant with large effect sizes; in the few samples exceeding these limits, the moderating effects’ confidence intervals gradually widened and approached the zero line. Young and middle-aged women (*n* = 372) followed similar moderating patterns, though their thresholds were approximately 3–4 g lower than those of men. The lower DSI moderating threshold reached 12.44 g for HDL-C (*p* = 0.0048, 98.92% below threshold), 12.15 g for UA (*p* = 0.0184, 98.39% below threshold), and 11.24 g for ApoA1 (*p* = 0.0140, 97.58% below threshold). These thresholds for women concentrated in the 11–12 g range, notably lower than the 13–16 g range observed in men, while moderating effects in the elderly population essentially disappeared (Table 8).

**Table 8:** Sensitivity analysis of moderating effects stratified by age and sex.

**Subgroup**	***N***	**HDL-C**	**UA**	**ApoA1**
		Lower threshold (*p*-value) [% of population]	Lower threshold (*p*-value) [% of population]	Lower threshold (*p*-value) [% of population]
Young and middle-aged Male	1,144	15.95 g (0.0012) [99.91%]	16.15 g (0.0042) [99.91%]	13.38 g (0.0007) [98.03%]
Young and middle-aged women	372	12.44 g (0.0048) [98.92%]	12.15 g (0.0184) [98.39%]	11.24 g (0.0140) [97.58%]
Elderly man	189	N/A (0.700)	N/A (0.776)	N/A (0.573)
Elderly woman	161	N/A (0.230)	N/A (0.825)	N/A (0.663)

Thresholds represent the lower critical DSI values (g/d) determined by J-N analysis; *P*-values in parentheses indicate the significance of BMI × DSI interaction terms; percentages in square brackets represent the proportion of the subgroup with DSI below the threshold; N/A indicates non-significant interaction terms; young and middle-aged are defined as <60 years, elderly as ≥60 years.

## 4 Discussion

Global health guidelines recommend limiting salt intake to 5 g/day (32). In the present study, Johnson–Neyman analysis identified moderating thresholds of daily salt intake between 11.24 g and 16.15 g. The associations between BMI and several key biomarkers were more evident at intake levels below these thresholds and became weaker at higher intake levels. This pattern suggests that the links among BMI, cardiometabolic markers, and DSI may not follow a simple linear pattern. The values reported here should not be taken as intake targets.

The pattern seen at lower DSI levels is consistent with previous work suggesting that the association between sodium exposure and health outcomes may be non-linear (32, 33). Recent large-scale analyses have also suggested that very low sodium intake may be linked to less favorable outcomes in some settings (34). In our cohort, the association between BMI and systemic dysfunction was more evident in the lower and moderate DSI groups. Although the cross-sectional design does not allow causal inference, the relationship between adiposity and metabolic disturbance may differ across levels of salt intake.

Compensatory neurohormonal responses to lower sodium intake may be involved in this pattern. Volume contraction can increase plasma renin and aldosterone activity (35), and aldosterone has been linked to insulin resistance and systemic inflammation independent of blood pressure (36). These processes are closely related to dyslipidemia and broader metabolic dysfunction associated with obesity. Meta-analyses of randomized trials have shown that sodium reduction lowers blood pressure, but may also be accompanied by changes in serum lipids (37). The stronger metabolic associations of higher BMI at lower DSI levels may partly reflect physiological responses to sodium reduction, although these pathways were not directly measured in the present study (38, 39).

The thresholds identified here, ranging from approximately 11–16 g, were higher than current domestic and international recommendations. This should not be interpreted as support for high salt intake. Within this cohort, the metabolic associations of excess body weight remained apparent across much of the observed salt intake range. Since most participants fell below the interaction thresholds, the metabolic burden associated with higher BMI may still be evident in the general working-age population. A single sodium restriction target may not fully capture differences in metabolic context (40–43).

The moderating effect was less apparent in the elderly group. Age-related arterial stiffening and declining renal function may reduce the contribution of dietary modulation to cardiometabolic variation in older adults (44). By contrast, younger adults showed clearer interaction patterns. We also observed sex differences in the thresholds, with lower values in women than in men. This is broadly consistent with previous evidence suggesting greater aldosterone responsiveness to sodium depletion in women (45) and may contribute to variation in how BMI-related metabolic associations appear across salt intake levels.

This study has several limitations that should be acknowledged when interpreting the findings. First, because of the cross-sectional design, causal inference cannot be established, and reverse causality cannot be excluded. Second, DSI was estimated from spot urine samples rather than repeated 24-h urine collections, which may have introduced measurement error, individual-level misclassification, and potential regression dilution bias (46). In addition, the absence of plasma renin activity or aldosterone measurements limited direct evaluation of the proposed neurohormonal mechanism. Residual confounding also remains possible. In particular, unmeasured lifestyle and dietary factors may have influenced both estimated DSI and cardiometabolic markers, thereby affecting the observed associations. The present findings should be interpreted with appropriate caution and further examined in future prospective and mechanistic studies.

## 5 Conclusion

In this Beijing occupational cohort, higher BMI was consistently associated with less favorable cardiometabolic profiles, and estimated DSI appeared to modify some of these associations, particularly for HDL-C and ApoA1, and mainly among adults younger than 60 years. However, the Johnson–Neyman thresholds identified here should be interpreted as statistical boundaries within this dataset rather than as recommended intake targets. Given the cross-sectional design and the use of spot urine-based estimation of salt intake, the present findings should be regarded as hypothesis-generating. Future prospective studies and intervention trials are needed to determine whether and how sodium intake modifies obesity-related metabolic risk in different populations.

## Data Availability

The raw data supporting the conclusions of this article will be made available by the authors, without undue reservation.
