# Supplementary material for: Salt intake moderates the association between BMI and cardiometabolic risk: evidence from an occupational cohort in Beijing
Source: Front Nutr. 2026 Apr 22;13:1815741. doi: 10.3389/fnut.2026.1815741 (PMC13144143; doi:10.3389/fnut.2026.1815741)
Supplement: Supplementary file 1 [file Data_Sheet_1.zip › (NEW)supplement 2.pdf]

Restricted Cubic Splines: BMI (kg/m²) vs ALT (U/L)

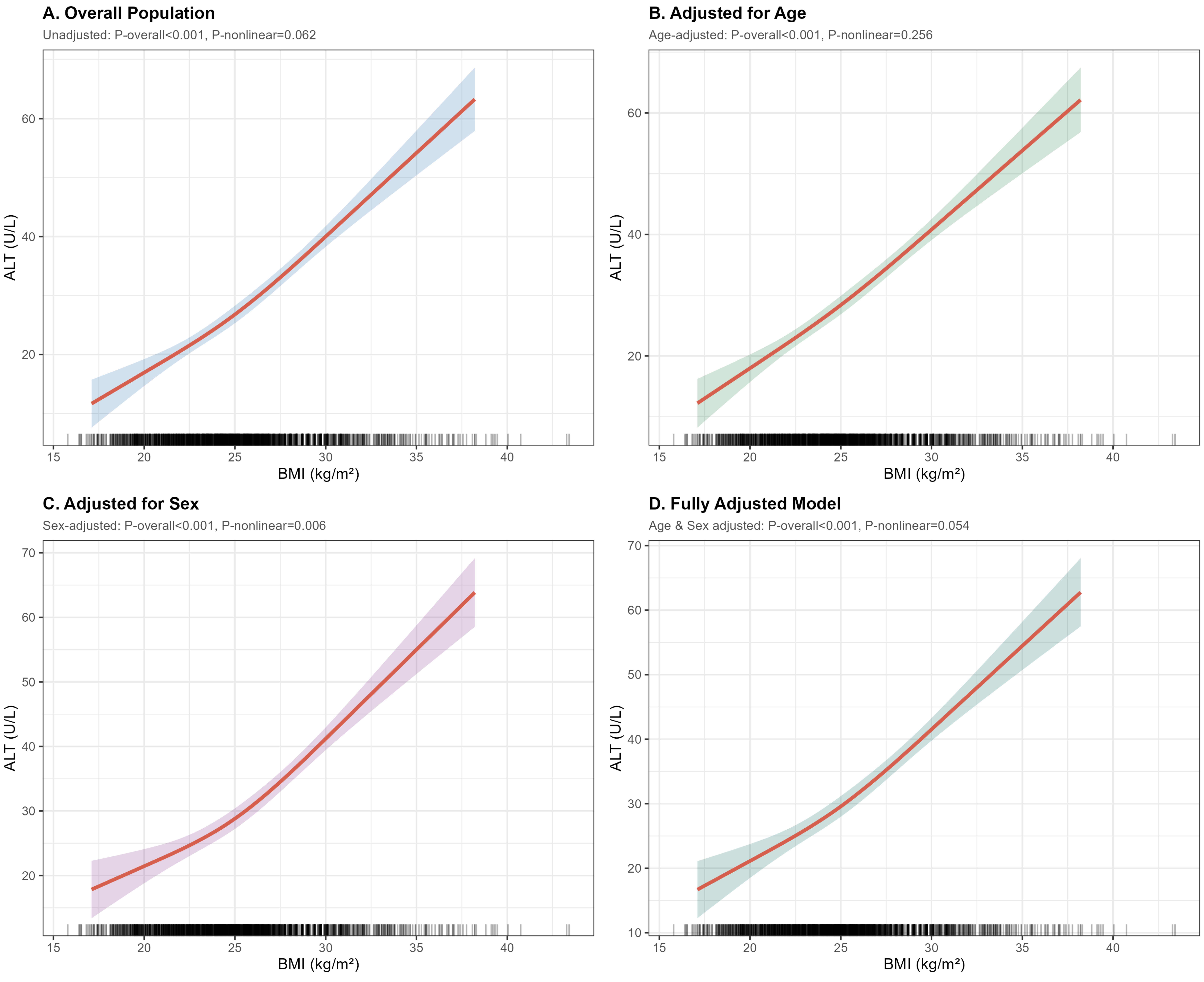

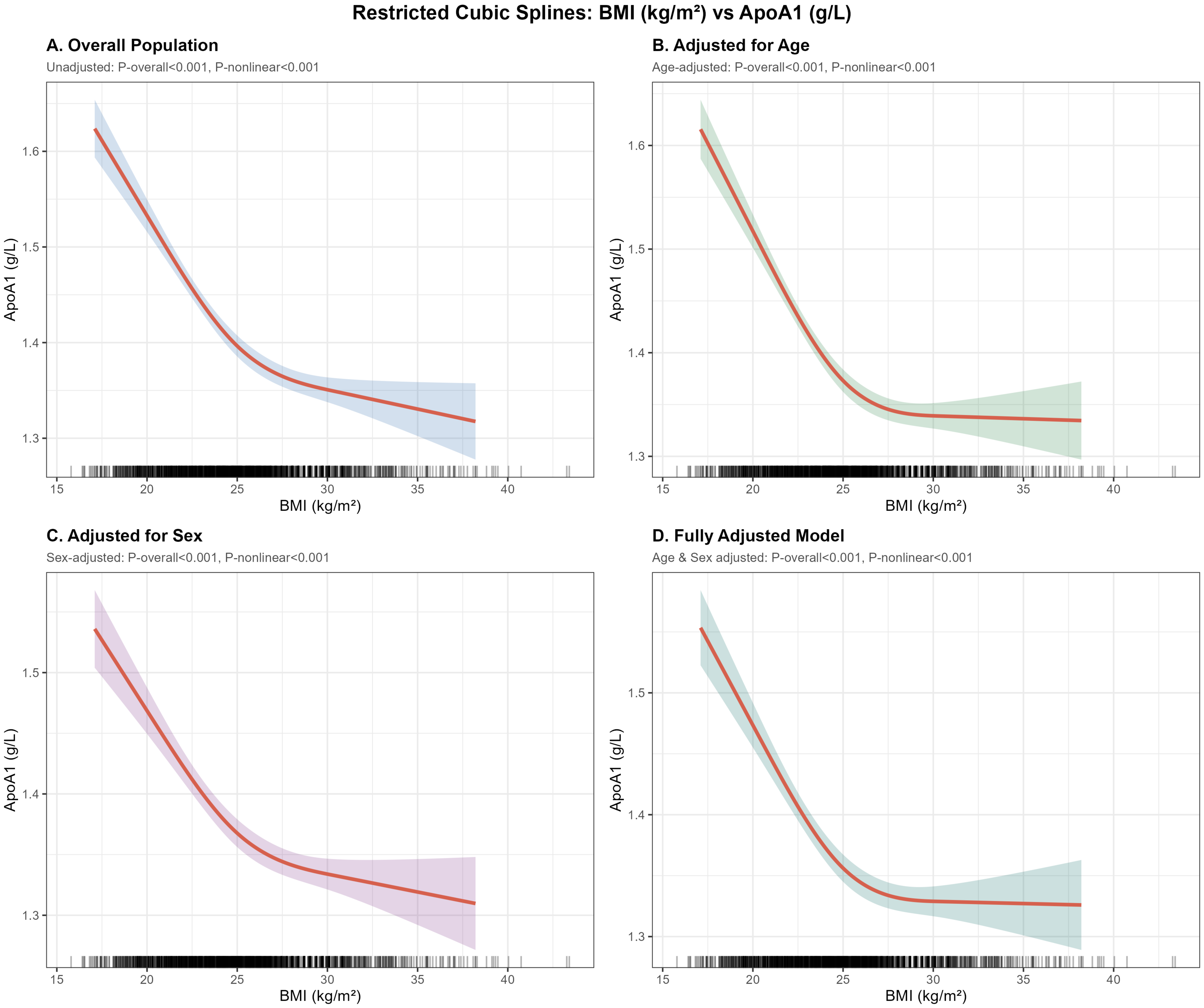
Restricted Cubic Splines: BMI (kg/m²) vs ApoA1 (g/L)
A. Overall Population
Unadjusted: P-overall<0.001, P-nonlinear<0.001
B. Adjusted for Age
Age-adjusted: P-overall<0.001, P-nonlinear<0.001
C. Adjusted for Sex
Sex-adjusted: P-overall<0.001, P-nonlinear<0.001
D. Fully Adjusted Model
Age & Sex adjusted: P-overall<0.001, P-nonlinear<0.001
ApoA1 (g/L)
BMI (kg/m²)
1.3
1.4
1.5
1.6
15
20
25
30
35
40

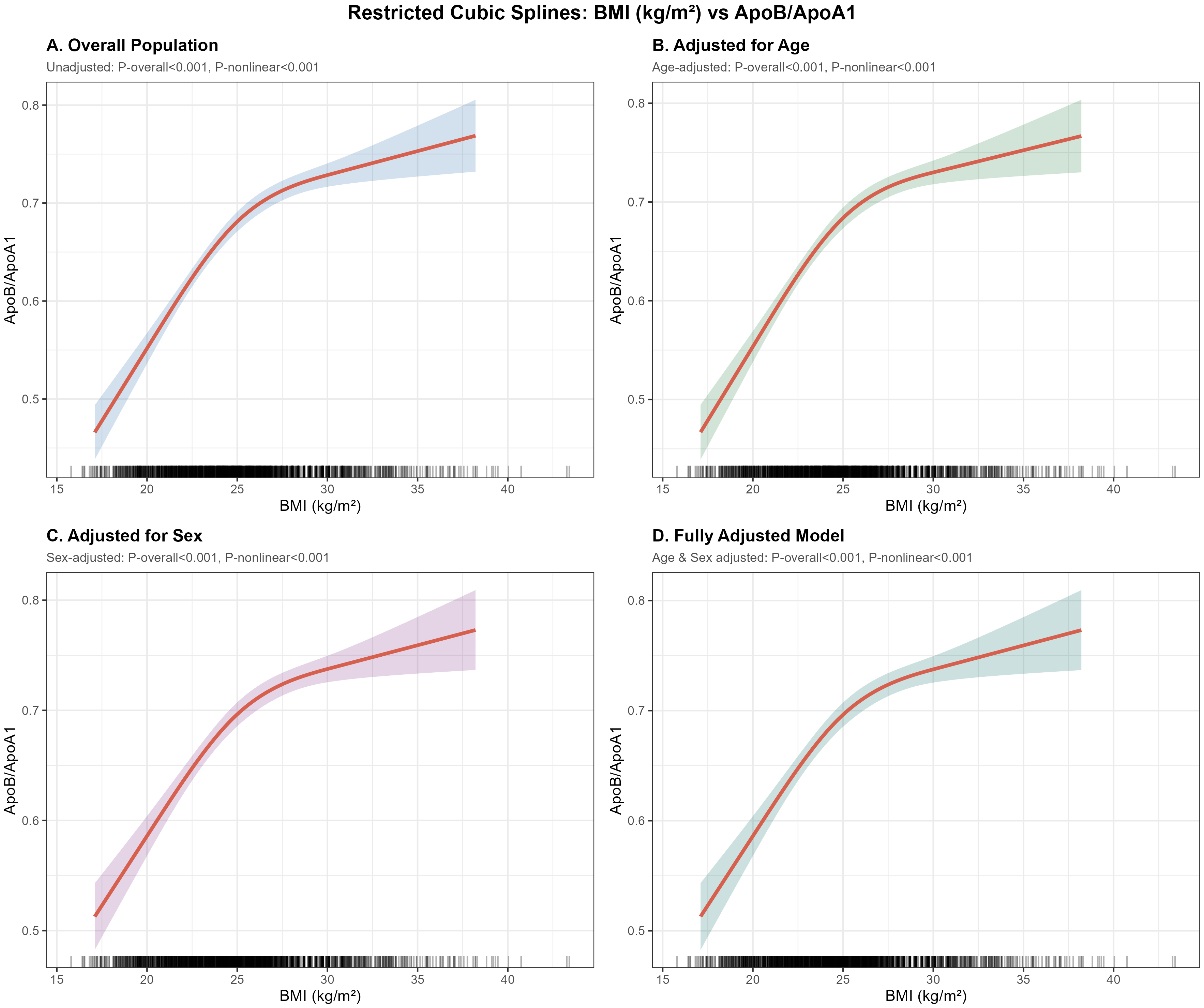
Restricted Cubic Splines: BMI (kg/m²) vs ApoB/ApoA1
A. Overall Population
Unadjusted: P-overall<0.001, P-nonlinear<0.001
B. Adjusted for Age
Age-adjusted: P-overall<0.001, P-nonlinear<0.001
C. Adjusted for Sex
Sex-adjusted: P-overall<0.001, P-nonlinear<0.001
D. Fully Adjusted Model
Age & Sex adjusted: P-overall<0.001, P-nonlinear<0.001
ApoB/ApoA1
BMI (kg/m²)
0.5
0.6
0.7
0.8
15
20
25
30
35
40

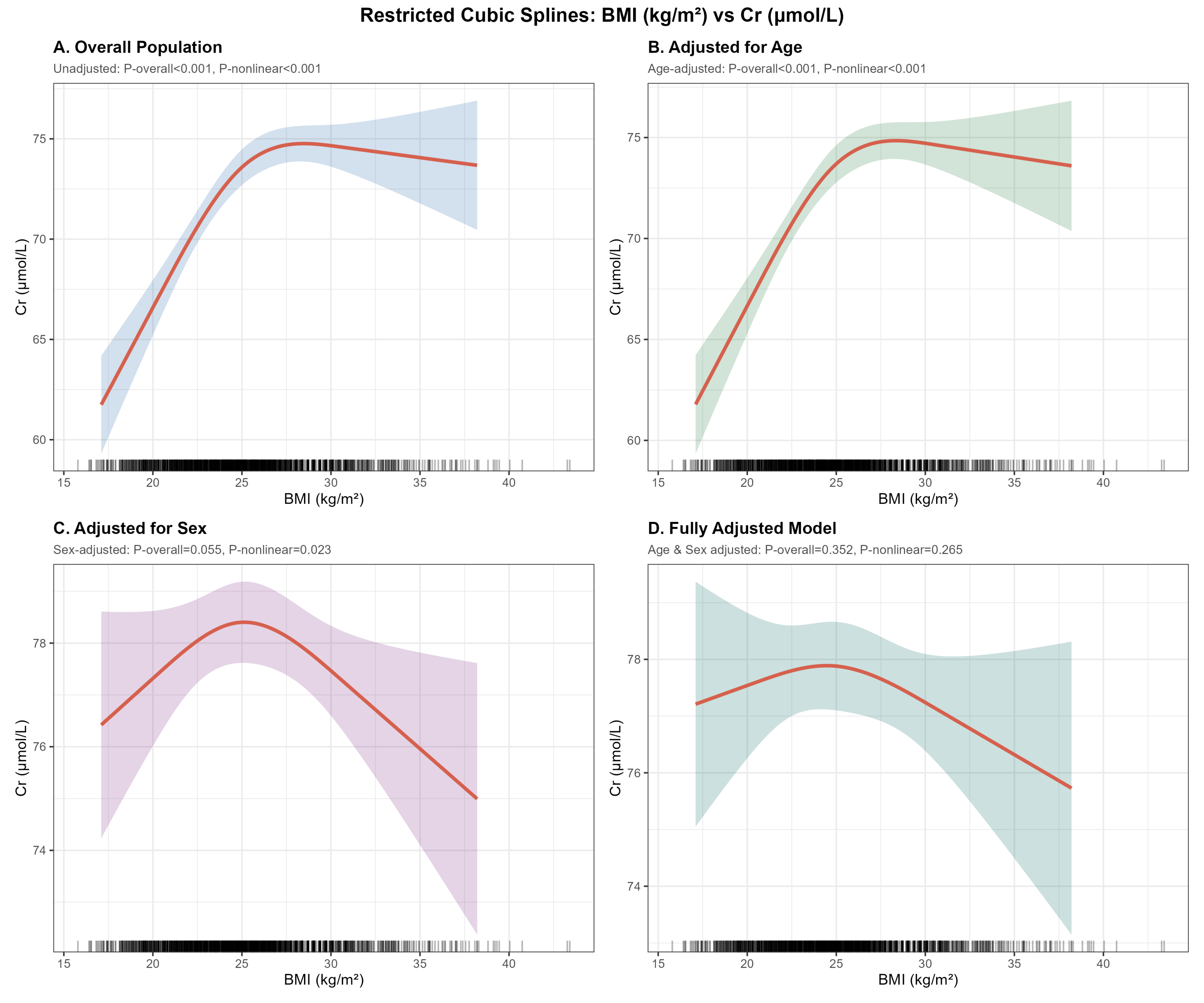

Restricted Cubic Splines: BMI (kg/m²) vs Cr (μmol/L)
A. Overall Population
Unadjusted: P-overall<0.001, P-nonlinear<0.001
B. Adjusted for Age
Age-adjusted: P-overall<0.001, P-nonlinear<0.001
C. Adjusted for Sex
Sex-adjusted: P-overall=0.055, P-nonlinear=0.023
D. Fully Adjusted Model
Age & Sex adjusted: P-overall=0.352, P-nonlinear=0.265
Cr (μmol/L)
BMI (kg/m²)
60
65
70
75
74
76
78
15
20
25
30
35
40

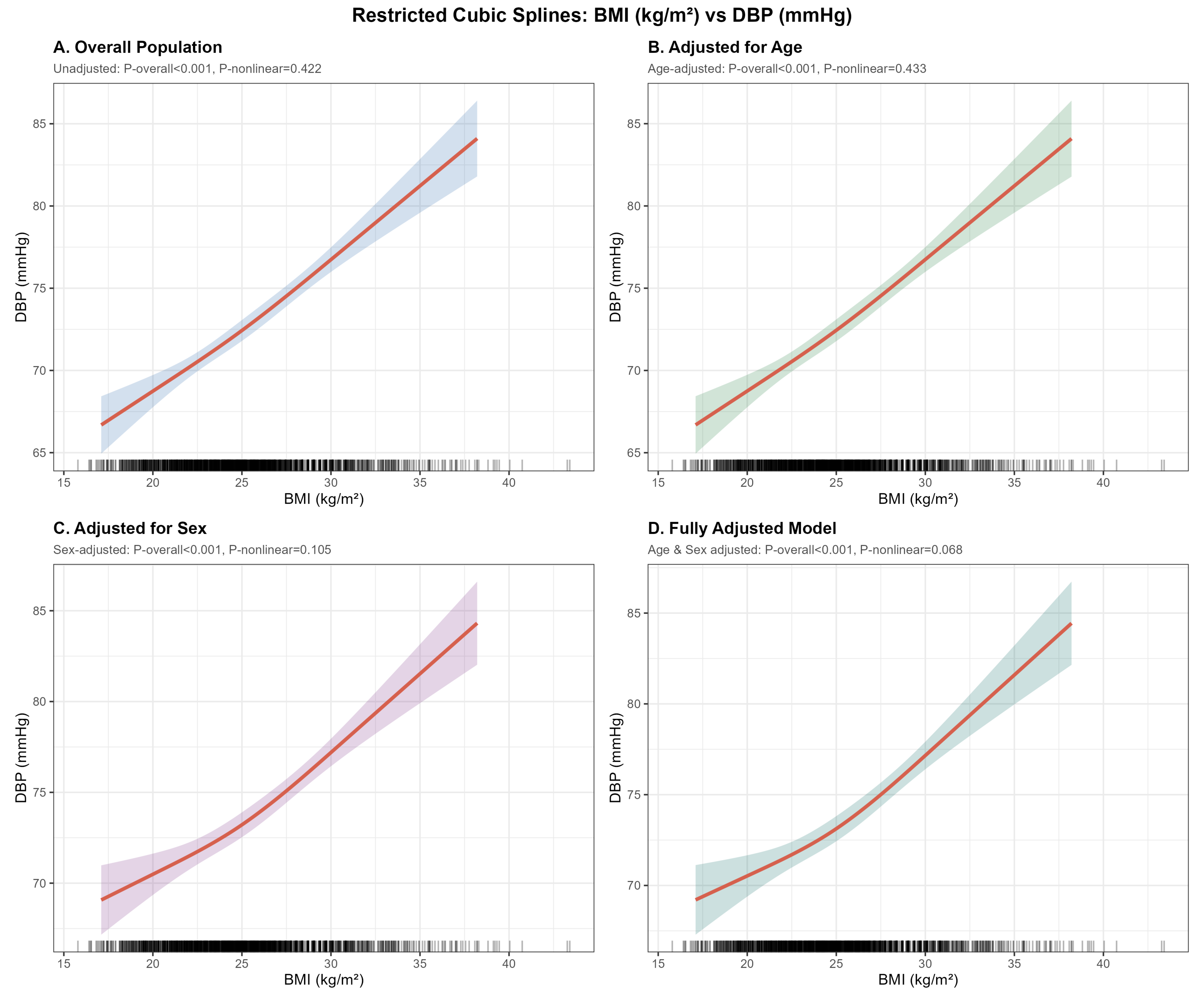

Restricted Cubic Splines: BMI (kg/m²) vs DBP (mmHg)
A. Overall Population
Unadjusted: P-overall<0.001, P-nonlinear=0.422
B. Adjusted for Age
Age-adjusted: P-overall<0.001, P-nonlinear=0.433
C. Adjusted for Sex
Sex-adjusted: P-overall<0.001, P-nonlinear=0.105
D. Fully Adjusted Model
Age & Sex adjusted: P-overall<0.001, P-nonlinear=0.068
DBP (mmHg)
BMI (kg/m²)
65
70
75
80
85
15
20
25
30
35
40

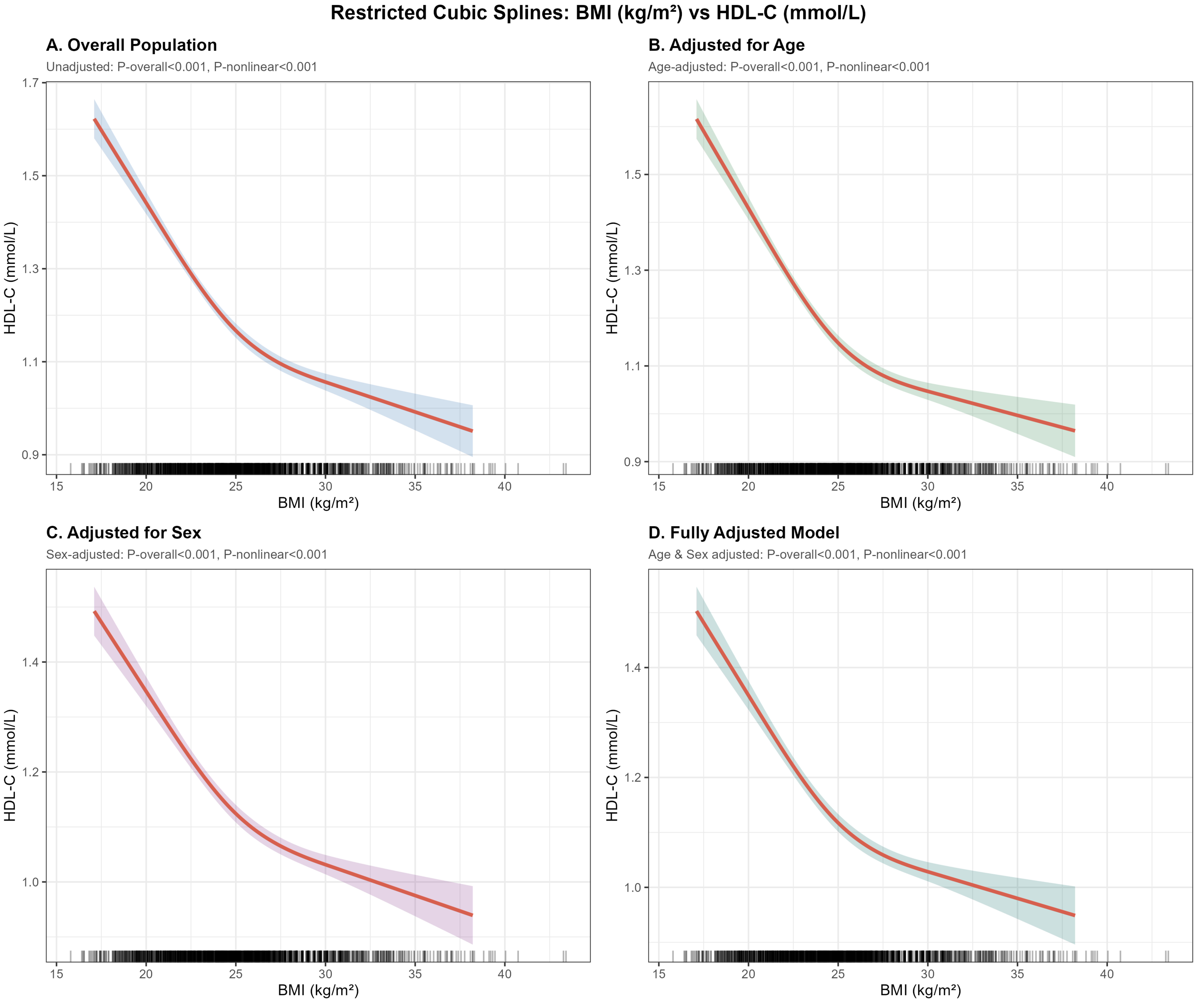

Restricted Cubic Splines: BMI (kg/m²) vs HDL-C (mmol/L)
A. Overall Population
Unadjusted: P-overall<0.001, P-nonlinear<0.001
B. Adjusted for Age
Age-adjusted: P-overall<0.001, P-nonlinear<0.001
C. Adjusted for Sex
Sex-adjusted: P-overall<0.001, P-nonlinear<0.001
D. Fully Adjusted Model
Age & Sex adjusted: P-overall<0.001, P-nonlinear<0.001
HDL-C (mmol/L)
BMI (kg/m²)
15
20
25
30
35
40
1.7
1.5
1.3
1.1
0.9
1.4
1.2
1.0

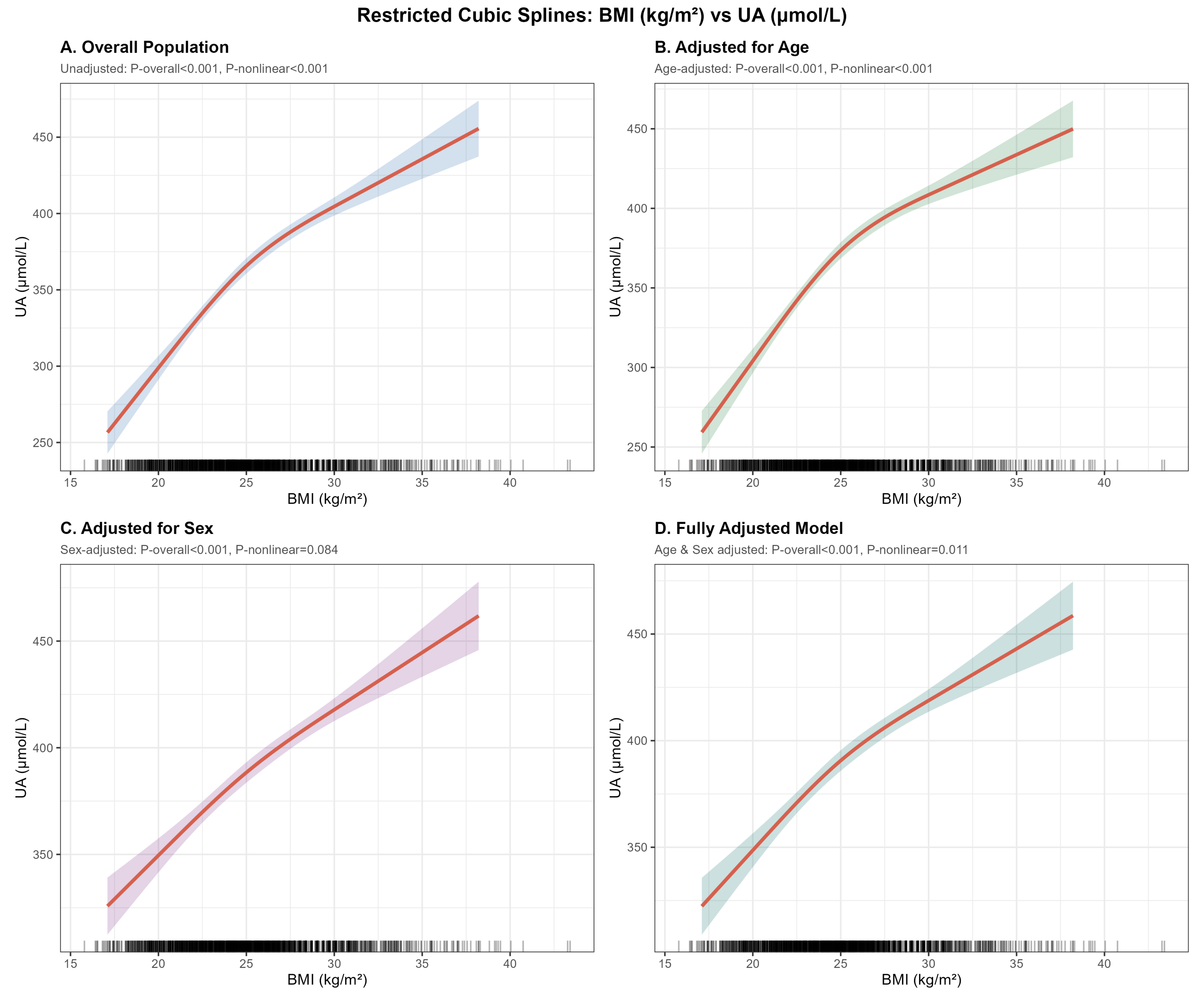

Restricted Cubic Splines: BMI (kg/m²) vs UA (μmol/L)
A. Overall Population
Unadjusted: P-overall<0.001, P-nonlinear<0.001
B. Adjusted for Age
Age-adjusted: P-overall<0.001, P-nonlinear<0.001
C. Adjusted for Sex
Sex-adjusted: P-overall<0.001, P-nonlinear=0.084
D. Fully Adjusted Model
Age & Sex adjusted: P-overall<0.001, P-nonlinear=0.011
UA (μmol/L)
BMI (kg/m²)

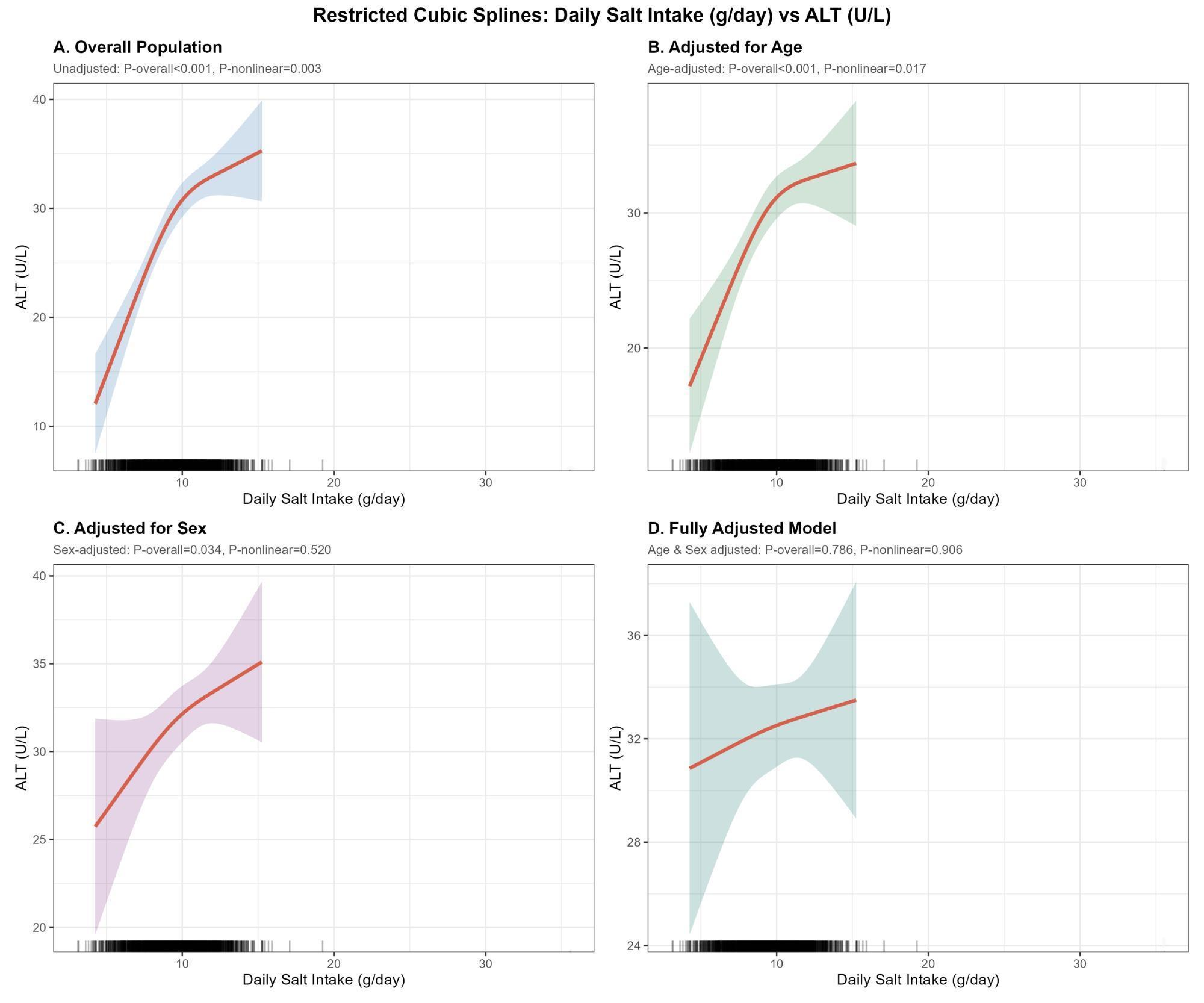
Restricted Cubic Splines: Daily Salt Intake (g/day) vs ALT (U/L)
A. Overall Population
Unadjusted: P-overall<0.001, P-nonlinear=0.003
B. Adjusted for Age
Age-adjusted: P-overall<0.001, P-nonlinear=0.017
C. Adjusted for Sex
Sex-adjusted: P-overall=0.034, P-nonlinear=0.520
D. Fully Adjusted Model
Age & Sex adjusted: P-overall=0.786, P-nonlinear=0.906
ALT (U/L)
Daily Salt Intake (g/day)
10
20
30
40
25
35
24
28
32
36

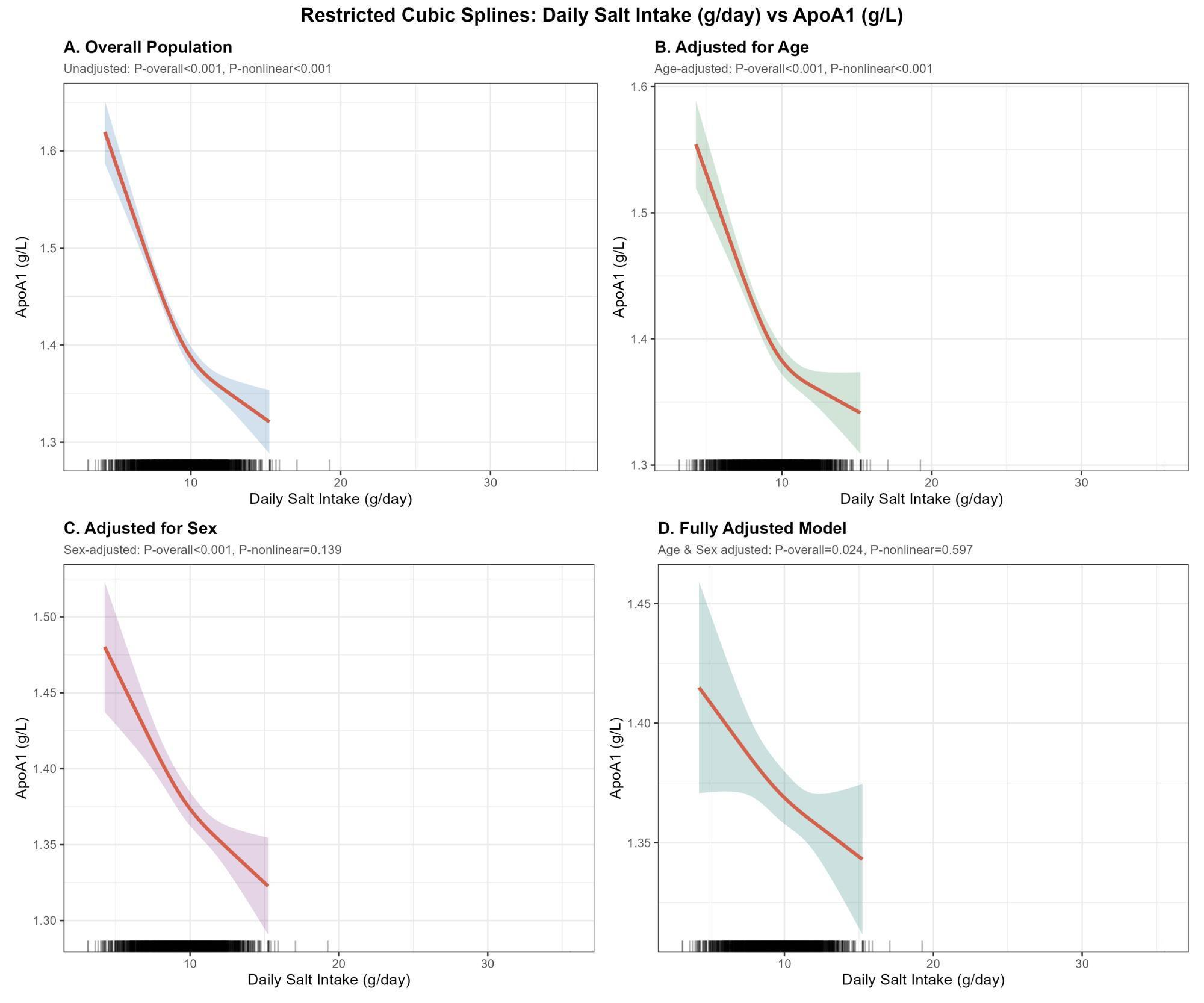

Restricted Cubic Splines: Daily Salt Intake (g/day) vs ApoA1 (g/L)
A. Overall Population
Unadjusted: P-overall<0.001, P-nonlinear<0.001
ApoA1 (g/L)
1.6
1.5
1.4
1.3
10
20
30
Daily Salt Intake (g/day)
B. Adjusted for Age
Age-adjusted: P-overall<0.001, P-nonlinear<0.001
ApoA1 (g/L)
1.6
1.5
1.4
1.3
10
20
30
Daily Salt Intake (g/day)
C. Adjusted for Sex
Sex-adjusted: P-overall<0.001, P-nonlinear=0.139
ApoA1 (g/L)
1.50
1.45
1.40
1.35
1.30
10
20
30
Daily Salt Intake (g/day)
D. Fully Adjusted Model
Age & Sex adjusted: P-overall=0.024, P-nonlinear=0.597
ApoA1 (g/L)
1.45
1.40
1.35
10
20
30
Daily Salt Intake (g/day)

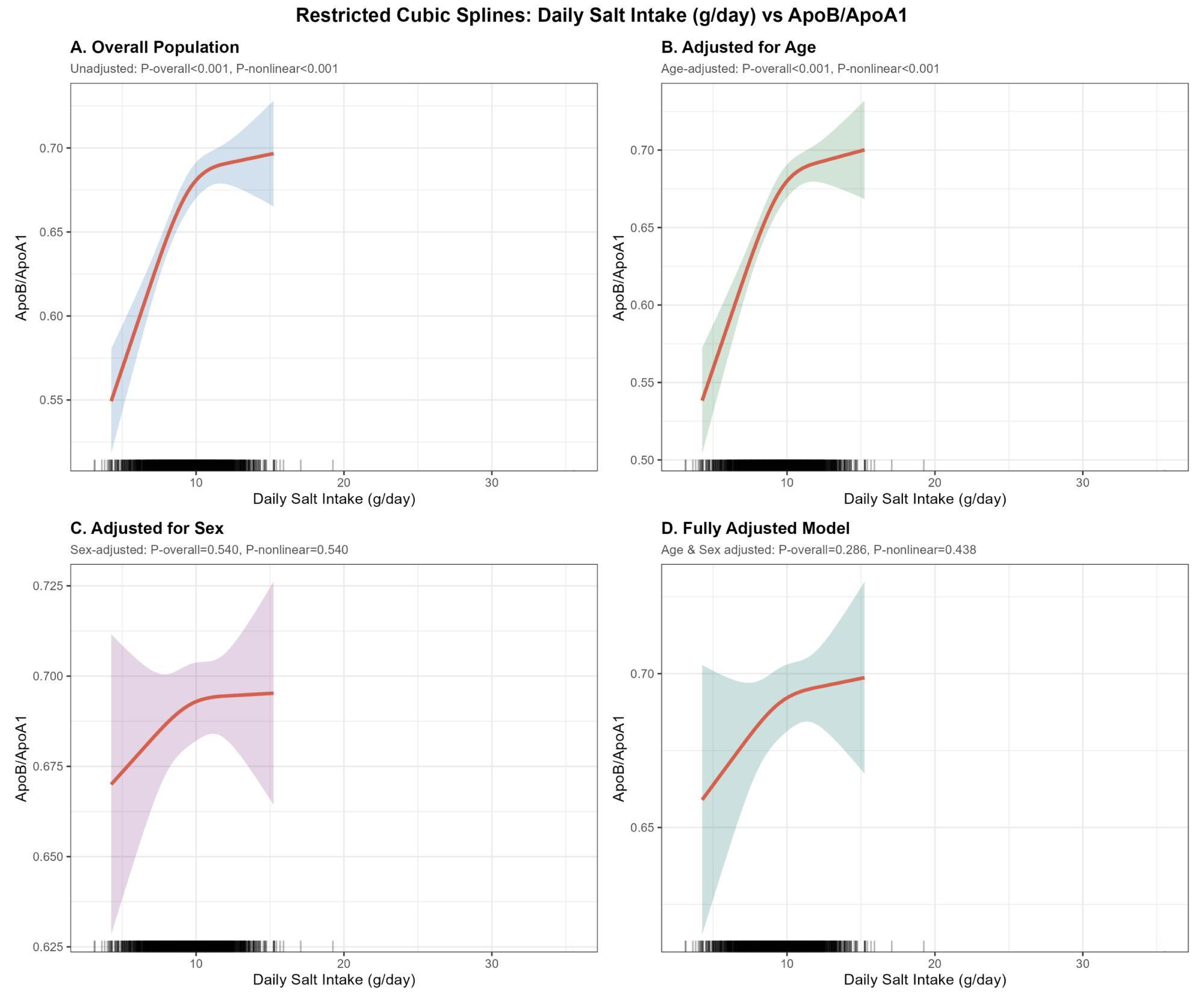
Restricted Cubic Splines: Daily Salt Intake (g/day) vs ApoB/ApoA1
A. Overall Population
Unadjusted: P-overall<0.001, P-nonlinear<0.001
B. Adjusted for Age
Age-adjusted: P-overall<0.001, P-nonlinear<0.001
C. Adjusted for Sex
Sex-adjusted: P-overall=0.540, P-nonlinear=0.540
D. Fully Adjusted Model
Age & Sex adjusted: P-overall=0.286, P-nonlinear=0.438
ApoB/ApoA1
Daily Salt Intake (g/day)
0.55
0.60
0.65
0.70
0.50
0.625
0.650
0.675
0.700
0.725
10
20
30

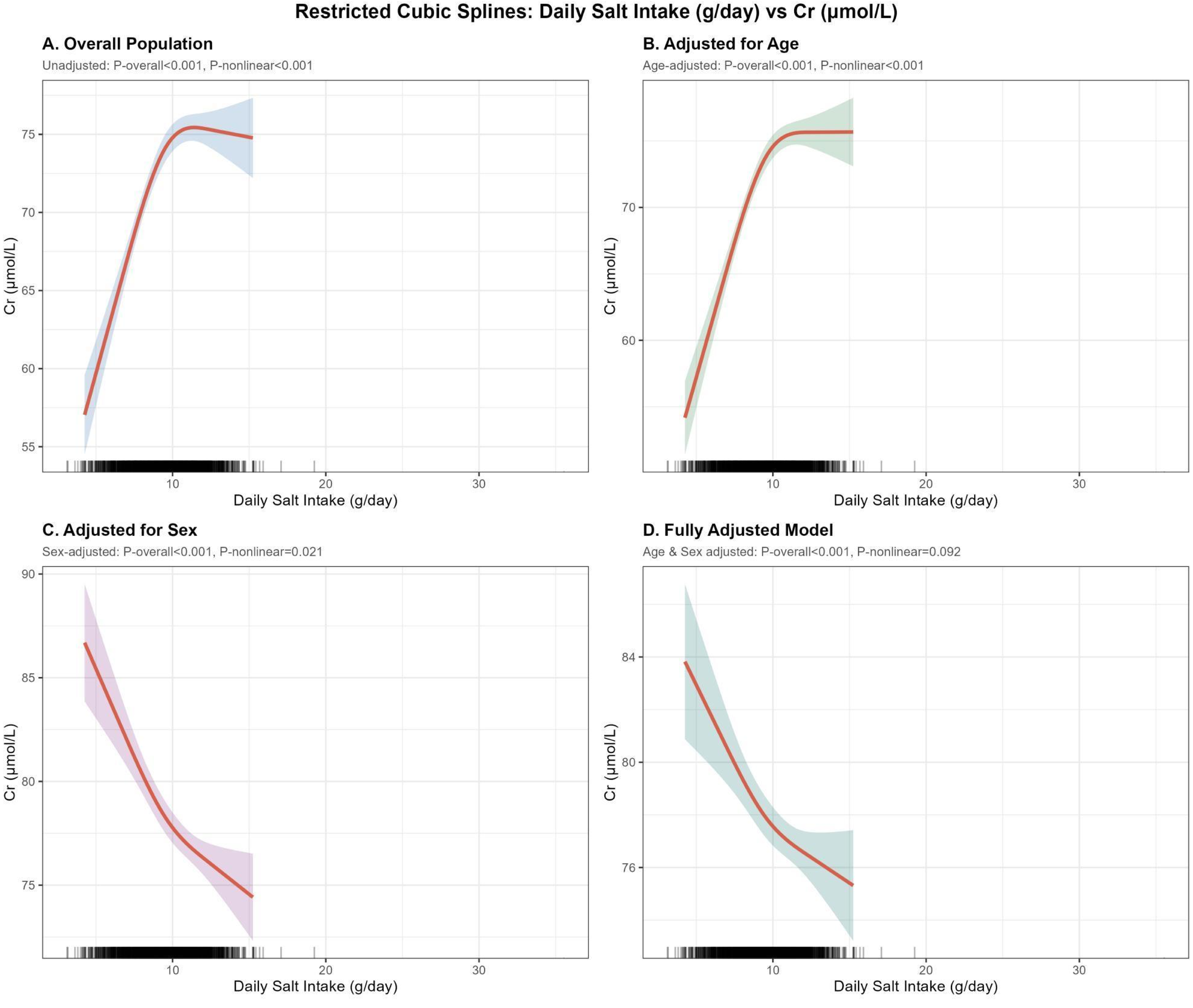

Restricted Cubic Splines: Daily Salt Intake (g/day) vs Cr (μmol/L)
A. Overall Population
Unadjusted: P-overall<0.001, P-nonlinear<0.001
B. Adjusted for Age
Age-adjusted: P-overall<0.001, P-nonlinear<0.001
C. Adjusted for Sex
Sex-adjusted: P-overall<0.001, P-nonlinear=0.021
D. Fully Adjusted Model
Age & Sex adjusted: P-overall<0.001, P-nonlinear=0.092
Cr (μmol/L)
Daily Salt Intake (g/day)
10
20
30
55
60
65
70
75
80
84
85
90
76

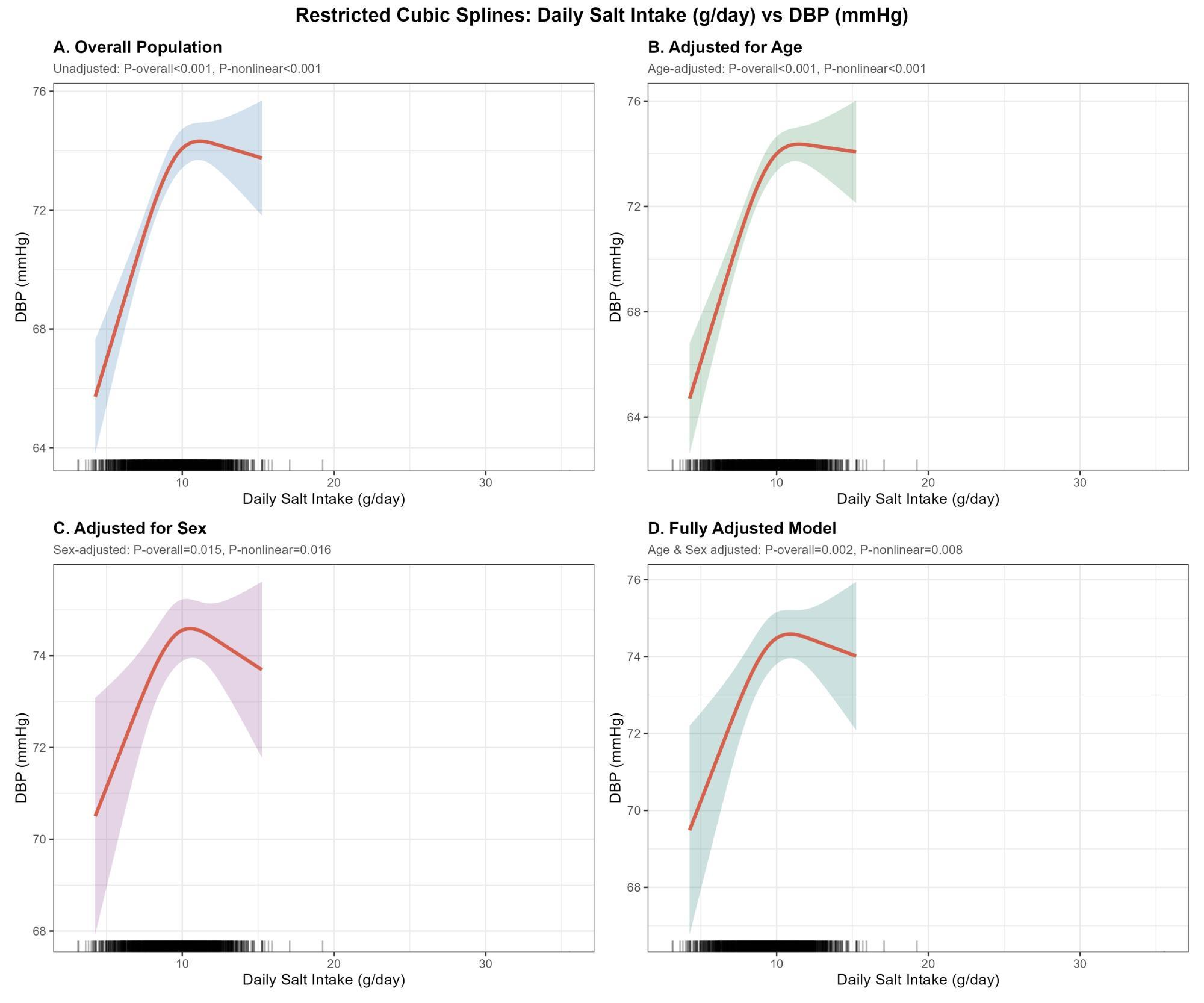

Restricted Cubic Splines: Daily Salt Intake (g/day) vs DBP (mmHg)
A. Overall Population
Unadjusted: P-overall<0.001, P-nonlinear<0.001
B. Adjusted for Age
Age-adjusted: P-overall<0.001, P-nonlinear<0.001
C. Adjusted for Sex
Sex-adjusted: P-overall=0.015, P-nonlinear=0.016
D. Fully Adjusted Model
Age & Sex adjusted: P-overall=0.002, P-nonlinear=0.008
DBP (mmHg)
Daily Salt Intake (g/day)
10
20
30
64
68
72
76
70
74

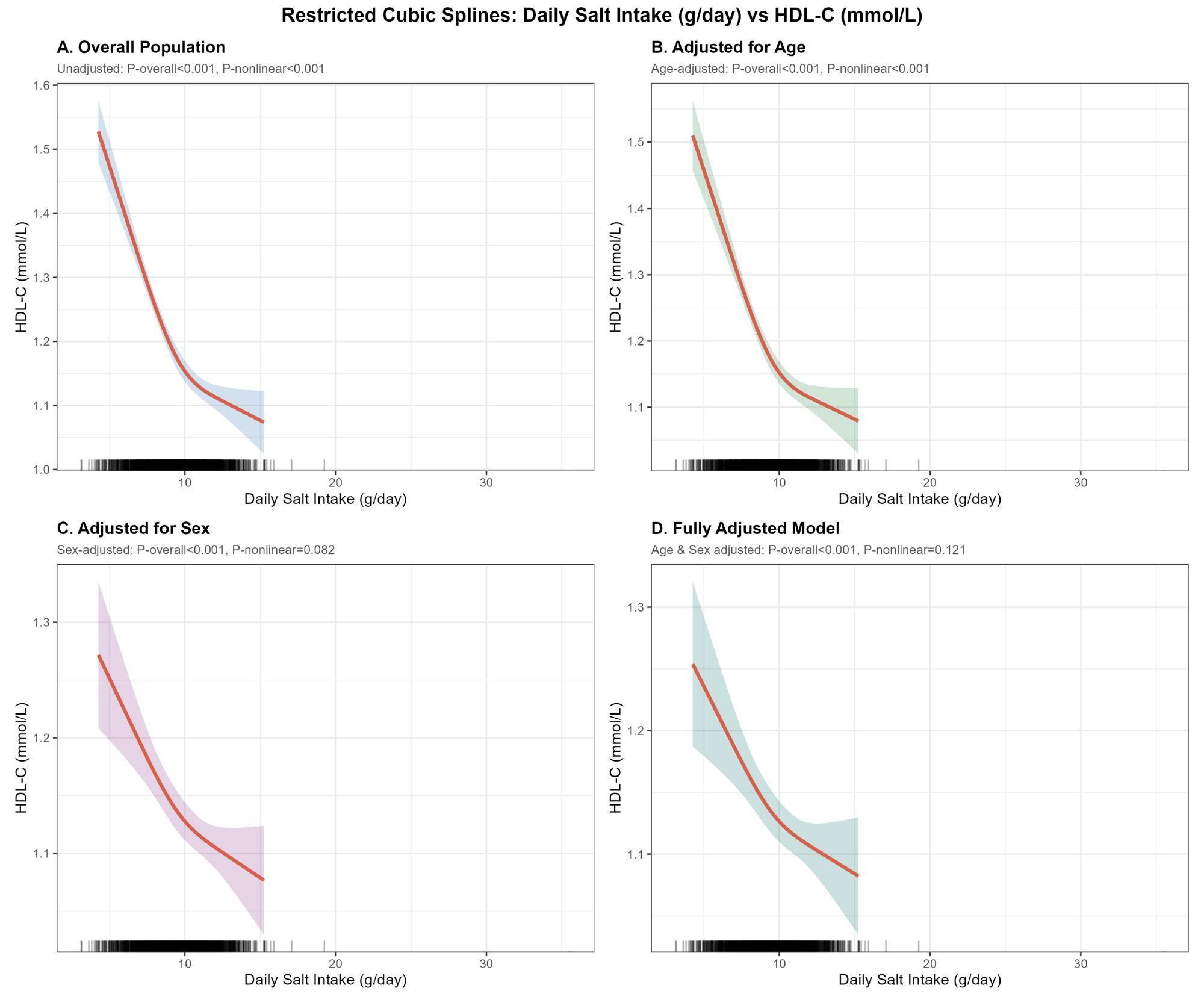
Restricted Cubic Splines: Daily Salt Intake (g/day) vs HDL-C (mmol/L)
A. Overall Population
Unadjusted: P-overall<0.001, P-nonlinear<0.001
B. Adjusted for Age
Age-adjusted: P-overall<0.001, P-nonlinear<0.001
C. Adjusted for Sex
Sex-adjusted: P-overall<0.001, P-nonlinear=0.082
D. Fully Adjusted Model
Age & Sex adjusted: P-overall<0.001, P-nonlinear=0.121
HDL-C (mmol/L)
Daily Salt Intake (g/day)
1.0
1.1
1.2
1.3
1.4
1.5
1.6
10
20
30

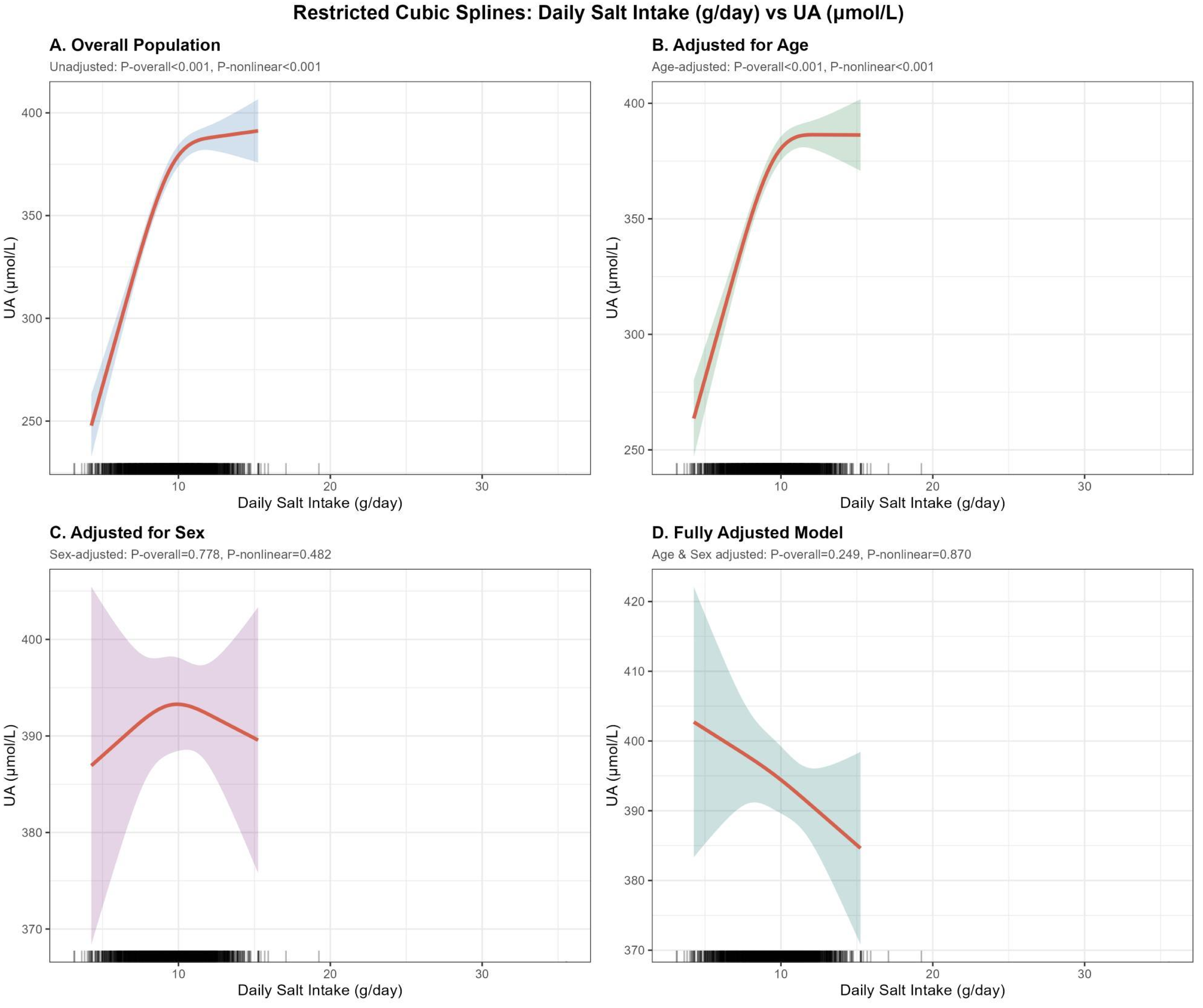
Restricted Cubic Splines: Daily Salt Intake (g/day) vs UA (μmol/L)
A. Overall Population
Unadjusted: P-overall<0.001, P-nonlinear<0.001
B. Adjusted for Age
Age-adjusted: P-overall<0.001, P-nonlinear<0.001
C. Adjusted for Sex
Sex-adjusted: P-overall=0.778, P-nonlinear=0.482
D. Fully Adjusted Model
Age & Sex adjusted: P-overall=0.249, P-nonlinear=0.870
UA (μmol/L)
Daily Salt Intake (g/day)
10
20
30
