# Supplementary material for: Salt intake moderates the association between BMI and cardiometabolic risk: evidence from an occupational cohort in Beijing
Source: Front Nutr. 2026 Apr 22;13:1815741. doi: 10.3389/fnut.2026.1815741 (PMC13144143; doi:10.3389/fnut.2026.1815741)
Supplement: Supplementary file 1 [file Data_Sheet_1.zip › (NEW)supplement 1.pdf]

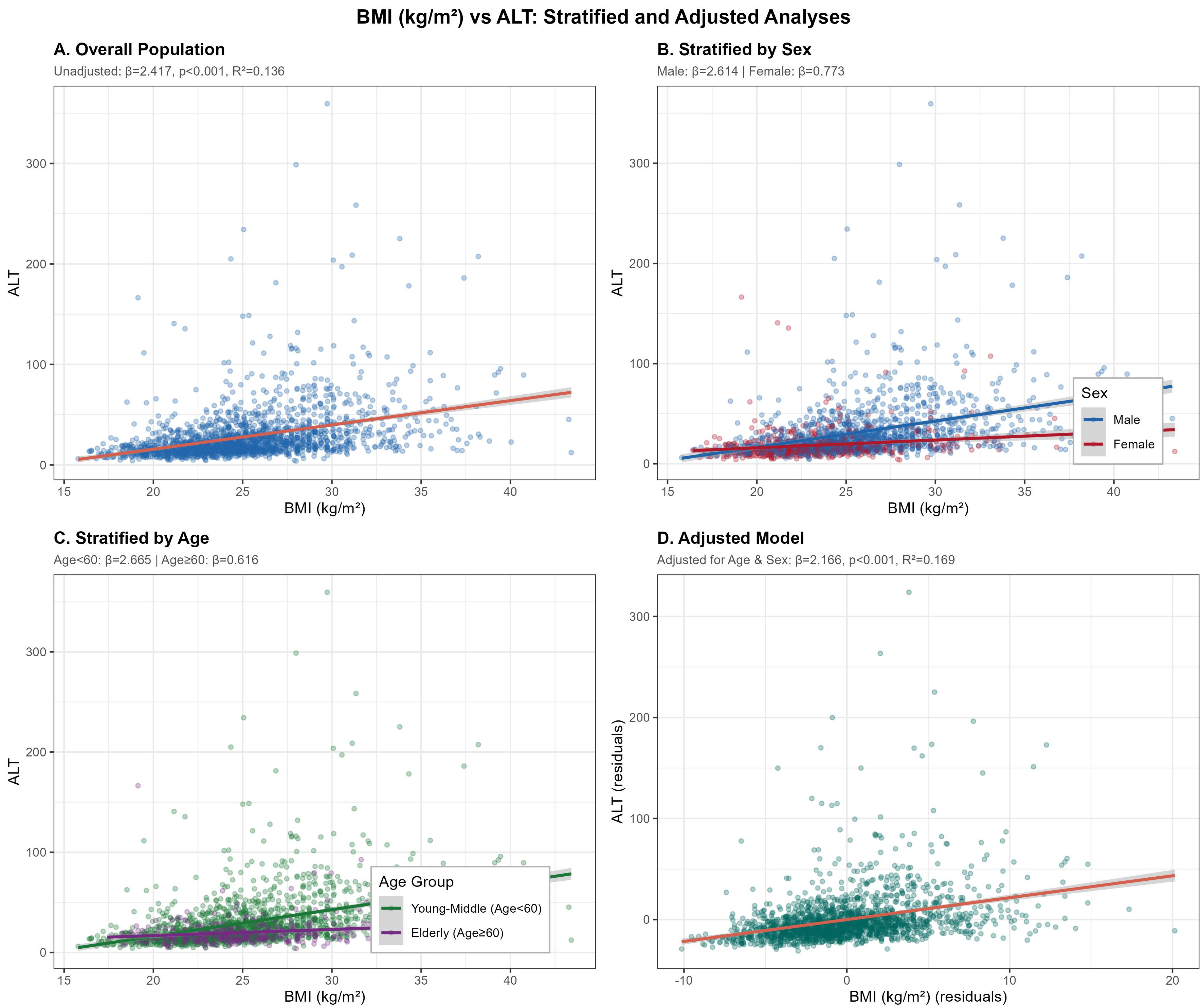
BMI (kg/m²) vs ALT: Stratified and Adjusted Analyses
A. Overall Population
Unadjusted: β=2.417, p<0.001, R²=0.136
ALT
BMI (kg/m²)
B. Stratified by Sex
Male: β=2.614 | Female: β=0.773
Sex
Male
Female
ALT
BMI (kg/m²)
C. Stratified by Age
Age<60: β=2.665 | Age≥60: β=0.616
Age Group
Young-Middle (Age<60)
Elderly (Age≥60)
ALT
BMI (kg/m²)
D. Adjusted Model
Adjusted for Age & Sex: β=2.166, p<0.001, R²=0.169
ALT (residuals)
BMI (kg/m²) (residuals)

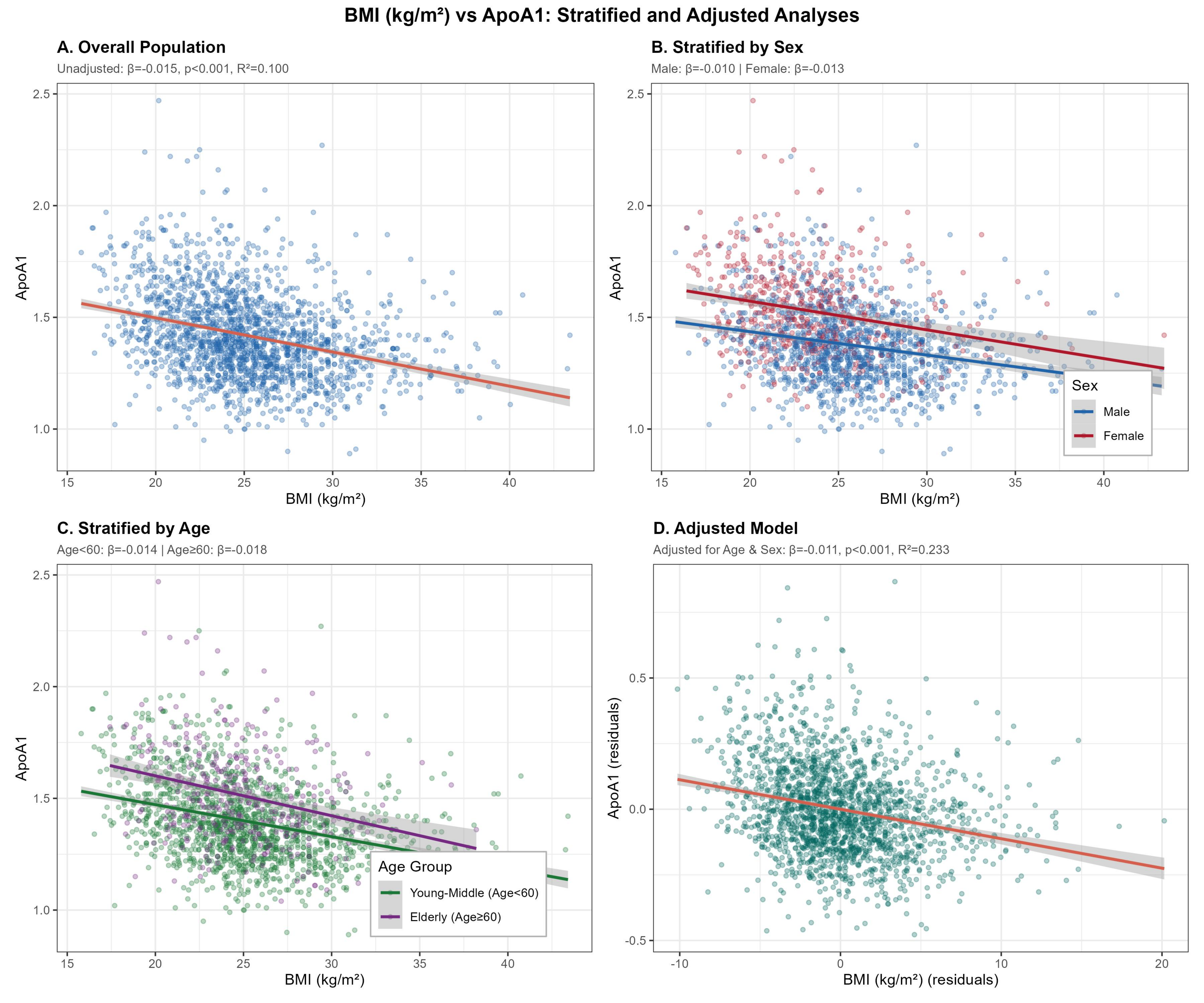

BMI (kg/m²) vs ApoA1: Stratified and Adjusted Analyses
A. Overall Population
Unadjusted: β=-0.015, p<0.001, R²=0.100
ApoA1
BMI (kg/m²)
B. Stratified by Sex
Male: β=-0.010 | Female: β=-0.013
Sex
Male
Female
C. Stratified by Age
Age<60: β=-0.014 | Age≥60: β=-0.018
Age Group
Young-Middle (Age<60)
Elderly (Age≥60)
D. Adjusted Model
Adjusted for Age & Sex: β=-0.011, p<0.001, R²=0.233
ApoA1 (residuals)
BMI (kg/m²) (residuals)

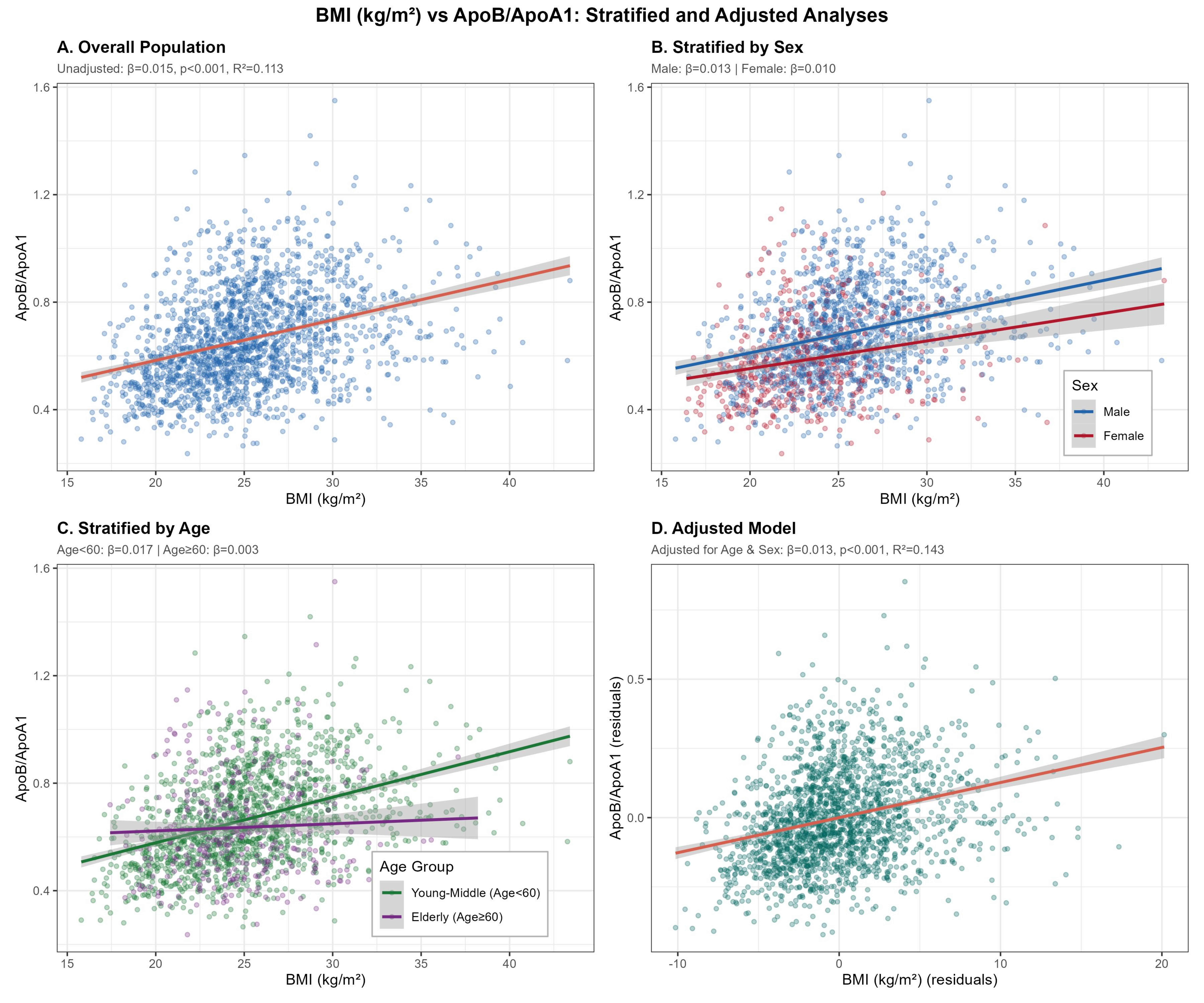
BMI (kg/m²) vs ApoB/ApoA1: Stratified and Adjusted Analyses
A. Overall Population
Unadjusted: β=0.015, p<0.001, R²=0.113
B. Stratified by Sex
Male: β=0.013 | Female: β=0.010
Sex
Male
Female
C. Stratified by Age
Age<60: β=0.017 | Age≥60: β=0.003
Age Group
Young-Middle (Age<60)
Elderly (Age≥60)
D. Adjusted Model
Adjusted for Age & Sex: β=0.013, p<0.001, R²=0.143
ApoB/ApoA1
BMI (kg/m²)
ApoB/ApoA1 (residuals)
BMI (kg/m²) (residuals)
1.6
1.2
0.8
0.4
15
20
25
30
35
40
0.5
0.0
-10
0
10
20

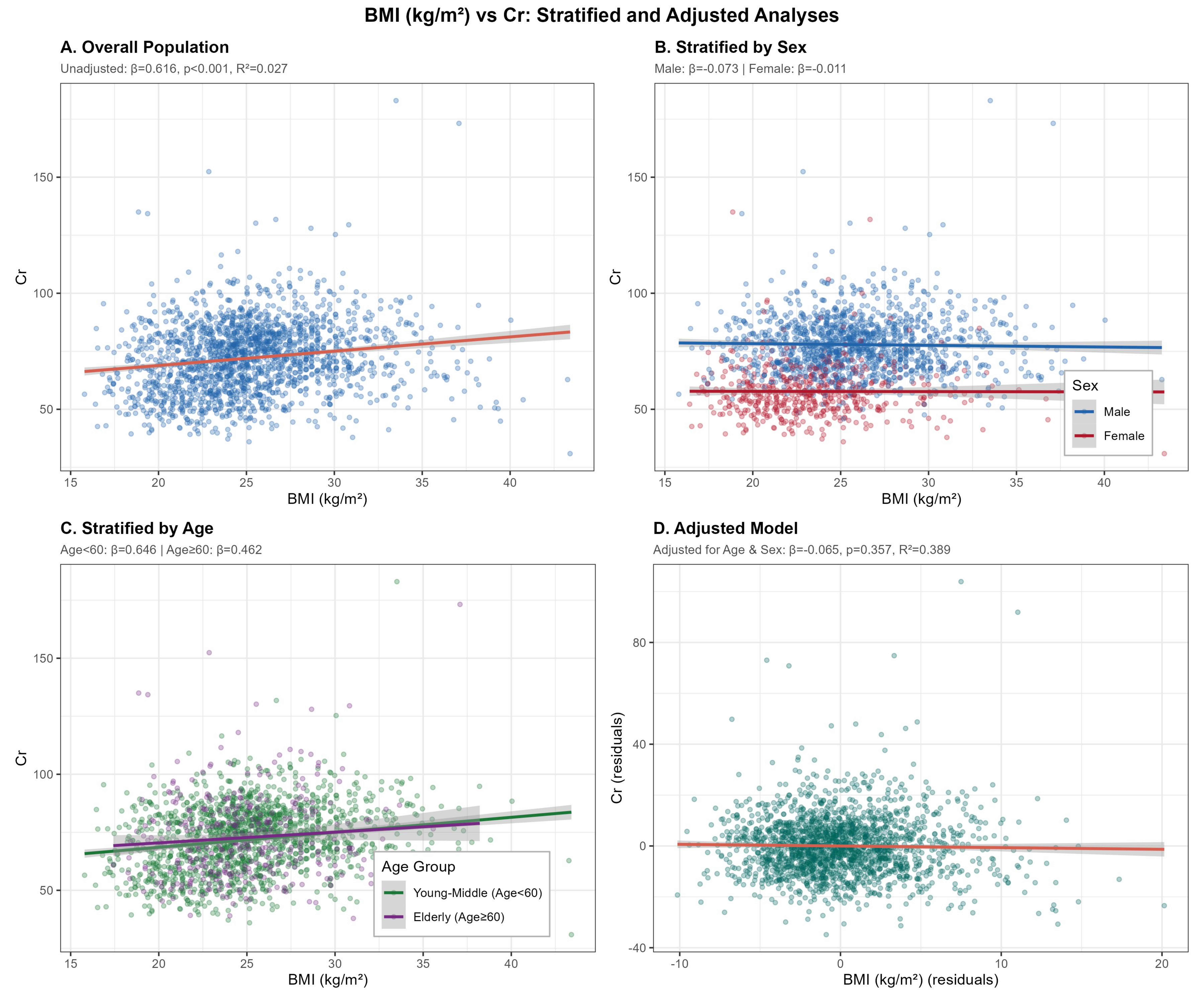
BMI (kg/m²) vs Cr: Stratified and Adjusted Analyses
A. Overall Population
Unadjusted: β=0.616, p<0.001, R²=0.027
Cr
BMI (kg/m²)
B. Stratified by Sex
Male: β=-0.073 | Female: β=-0.011
Sex
Male
Female
C. Stratified by Age
Age<60: β=0.646 | Age≥60: β=0.462
Age Group
Young-Middle (Age<60)
Elderly (Age≥60)
D. Adjusted Model
Adjusted for Age & Sex: β=-0.065, p=0.357, R²=0.389
Cr (residuals)
BMI (kg/m²) (residuals)

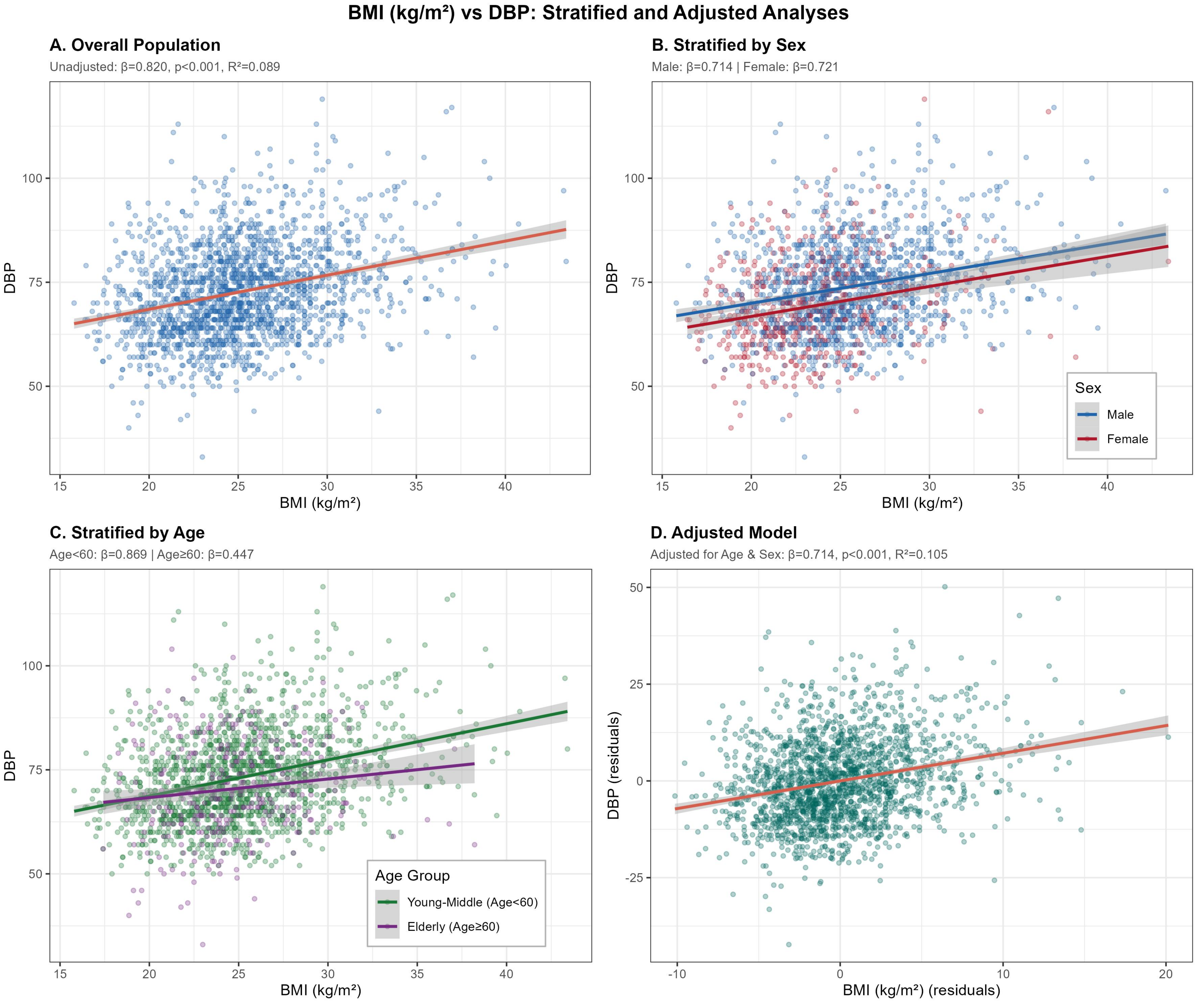

BMI (kg/m²) vs DBP: Stratified and Adjusted Analyses
A. Overall Population
Unadjusted: β=0.820, p<0.001, R²=0.089
DBP
BMI (kg/m²)
B. Stratified by Sex
Male: β=0.714 | Female: β=0.721
Sex
Male
Female
C. Stratified by Age
Age<60: β=0.869 | Age≥60: β=0.447
Age Group
Young-Middle (Age<60)
Elderly (Age≥60)
D. Adjusted Model
Adjusted for Age & Sex: β=0.714, p<0.001, R²=0.105
DBP (residuals)
BMI (kg/m²) (residuals)

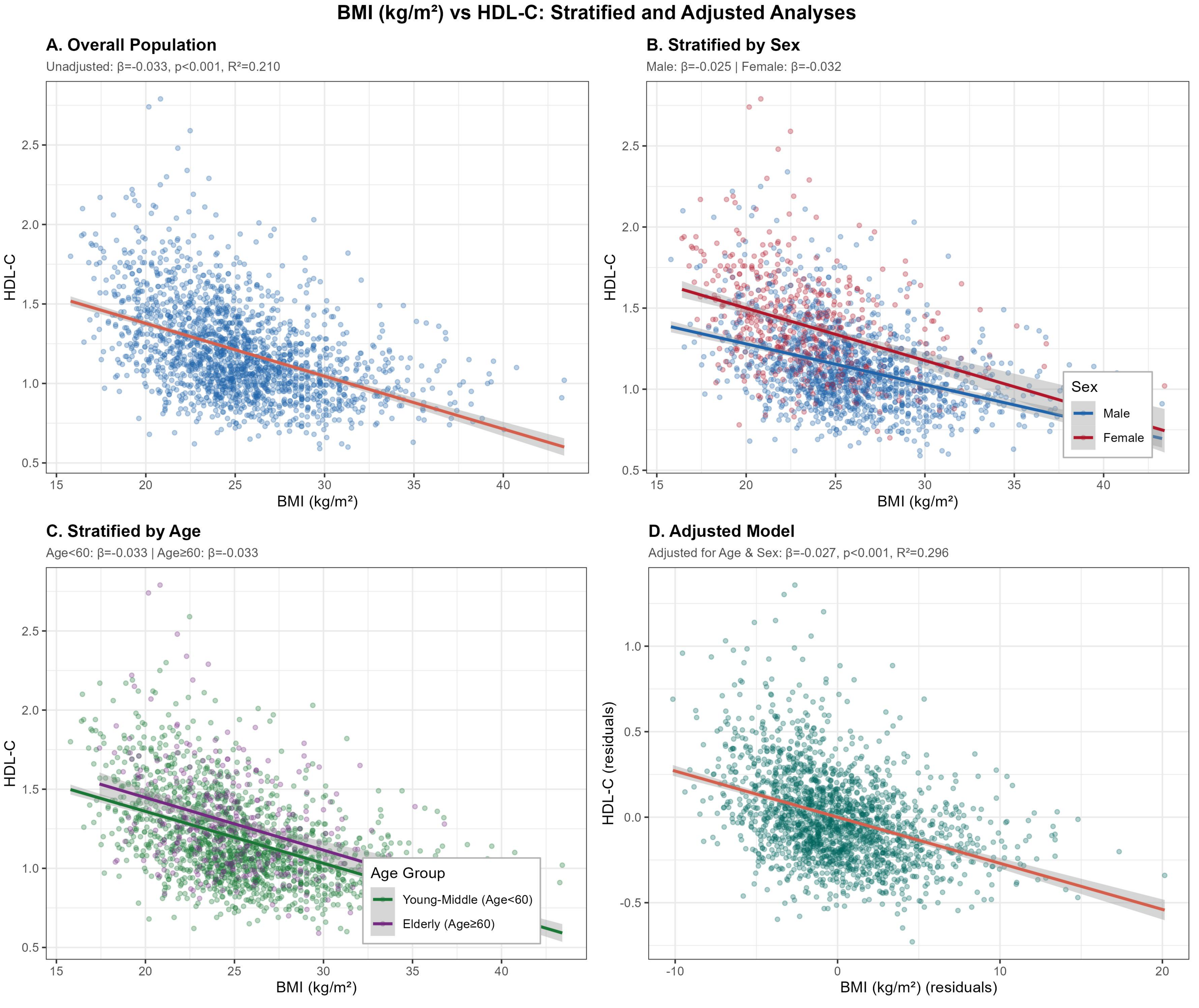
BMI (kg/m²) vs HDL-C: Stratified and Adjusted Analyses
A. Overall Population
Unadjusted: β=-0.033, p<0.001, R²=0.210
HDL-C
BMI (kg/m²)
B. Stratified by Sex
Male: β=-0.025 | Female: β=-0.032
Sex
Male
Female
C. Stratified by Age
Age<60: β=-0.033 | Age≥60: β=-0.033
Age Group
Young-Middle (Age<60)
Elderly (Age≥60)
D. Adjusted Model
Adjusted for Age & Sex: β=-0.027, p<0.001, R²=0.296
HDL-C (residuals)
BMI (kg/m²) (residuals)

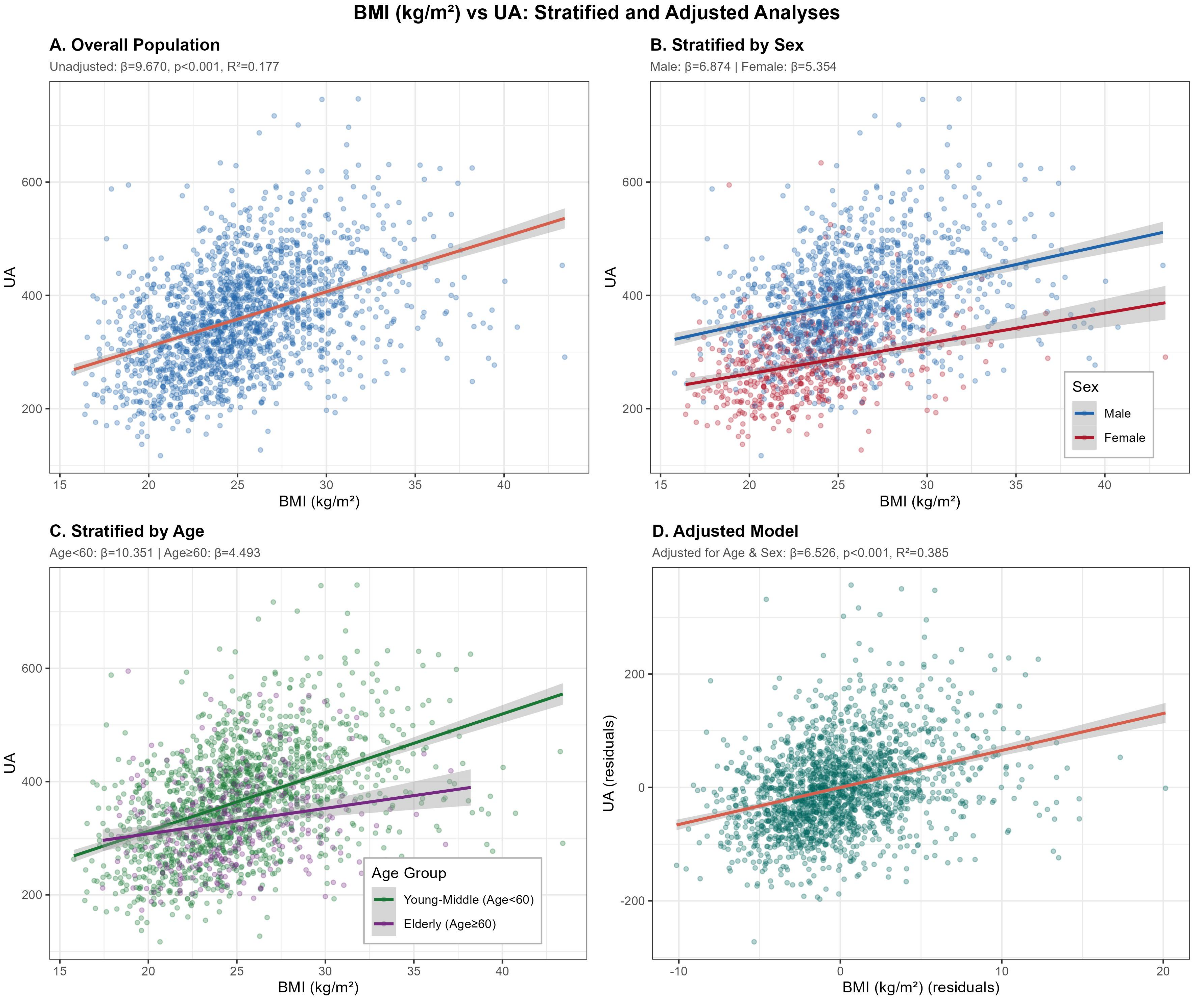
BMI (kg/m²) vs UA: Stratified and Adjusted Analyses
A. Overall Population
Unadjusted: β=9.670, p<0.001, R²=0.177
UA
BMI (kg/m²)
B. Stratified by Sex
Male: β=6.874 | Female: β=5.354
Sex
Male
Female
C. Stratified by Age
Age<60: β=10.351 | Age≥60: β=4.493
Age Group
Young-Middle (Age<60)
Elderly (Age≥60)
D. Adjusted Model
Adjusted for Age & Sex: β=6.526, p<0.001, R²=0.385
UA (residuals)
BMI (kg/m²) (residuals)

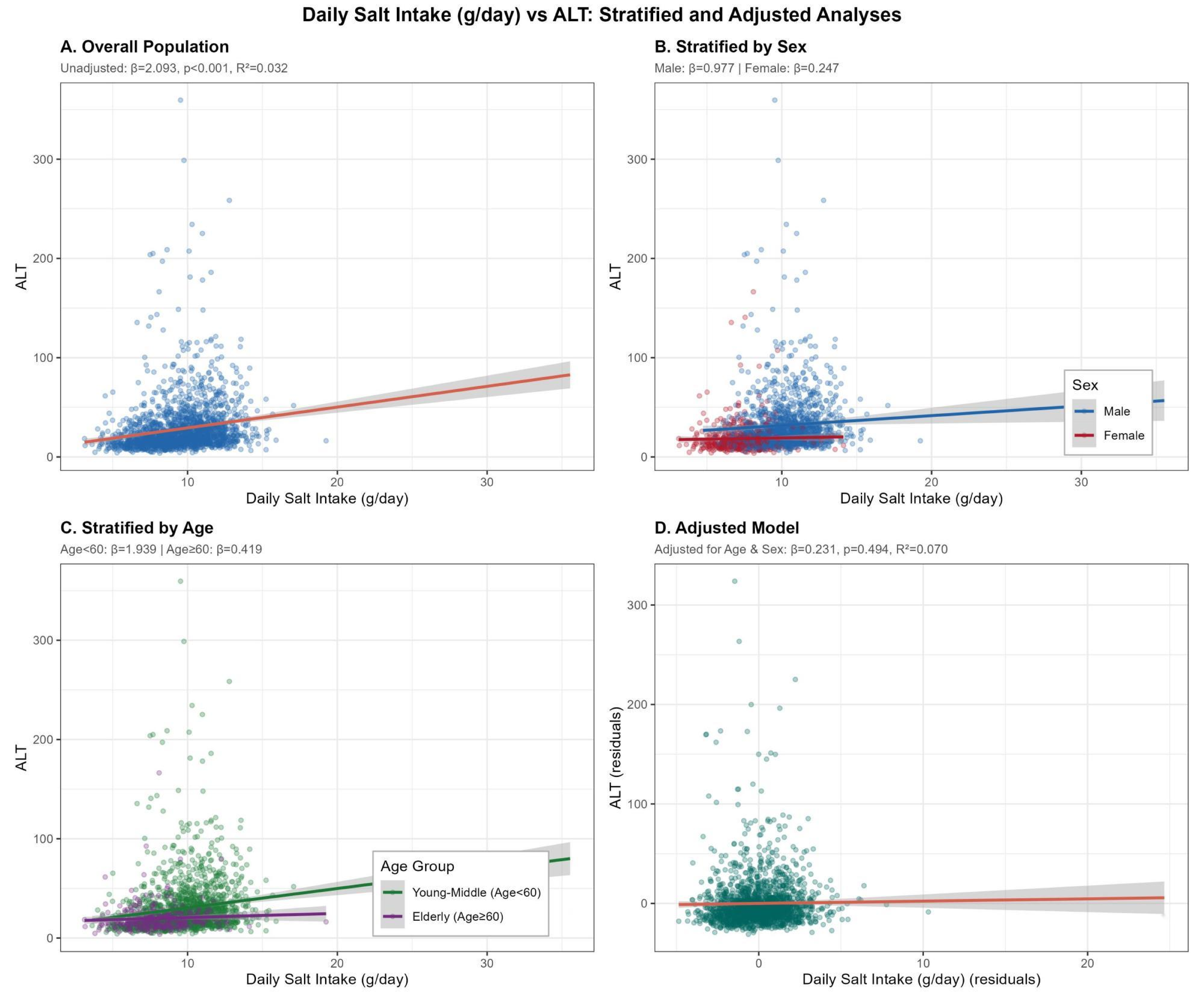

Daily Salt Intake (g/day) vs ALT: Stratified and Adjusted Analyses
A. Overall Population
Unadjusted: β=2.093, p<0.001, R²=0.032
ALT
Daily Salt Intake (g/day)
B. Stratified by Sex
Male: β=0.977 | Female: β=0.247
Sex
Male
Female
C. Stratified by Age
Age<60: β=1.939 | Age≥60: β=0.419
Age Group
Young-Middle (Age<60)
Elderly (Age≥60)
D. Adjusted Model
Adjusted for Age & Sex: β=0.231, p=0.494, R²=0.070
ALT (residuals)
Daily Salt Intake (g/day) (residuals)

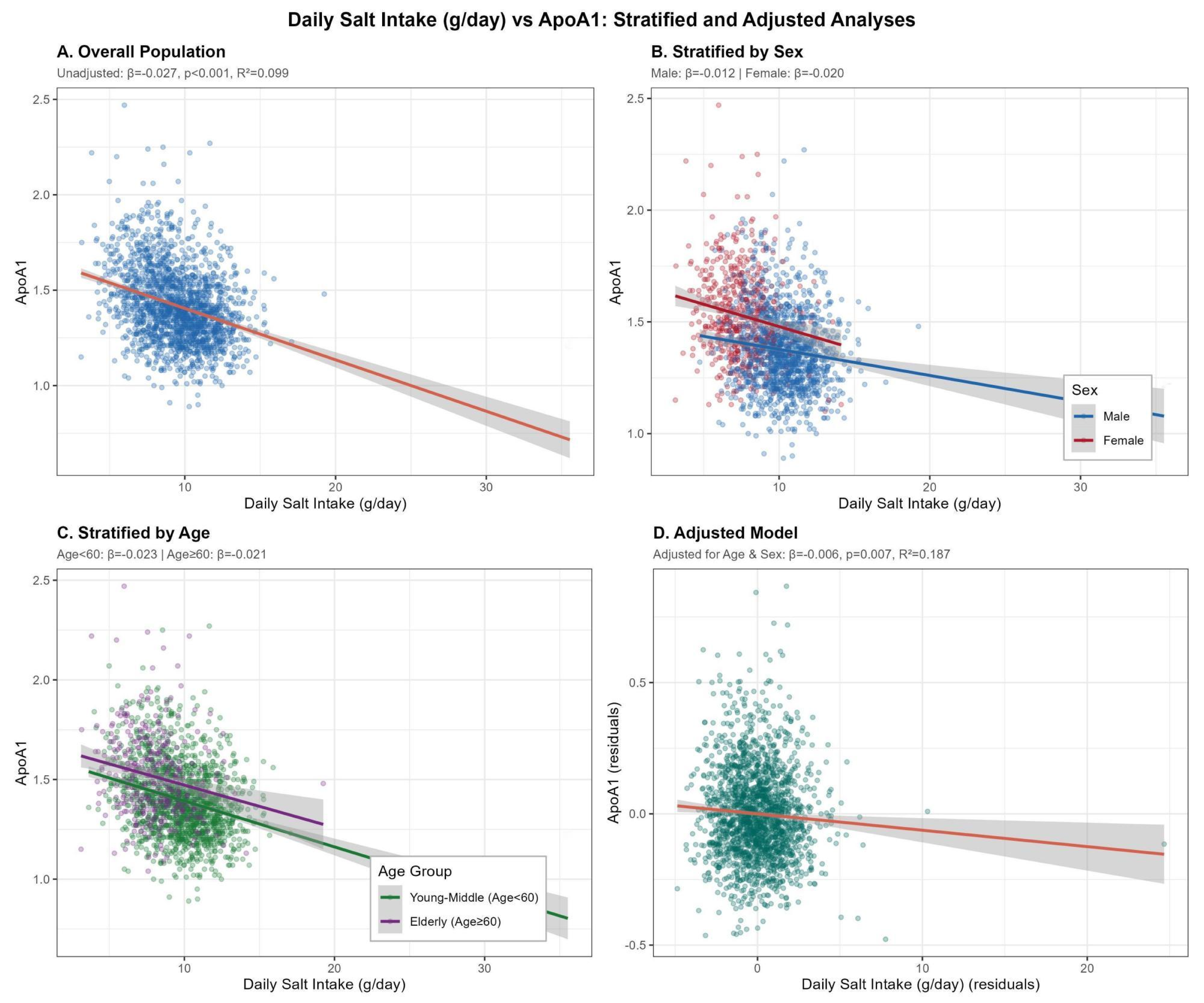
Daily Salt Intake (g/day) vs ApoA1: Stratified and Adjusted Analyses
A. Overall Population
Unadjusted: β=-0.027, p<0.001, R²=0.099
ApoA1
Daily Salt Intake (g/day)
B. Stratified by Sex
Male: β=-0.012 | Female: β=-0.020
Sex
Male
Female
C. Stratified by Age
Age<60: β=-0.023 | Age≥60: β=-0.021
Age Group
Young-Middle (Age<60)
Elderly (Age≥60)
D. Adjusted Model
Adjusted for Age & Sex: β=-0.006, p=0.007, R²=0.187
ApoA1 (residuals)
Daily Salt Intake (g/day) (residuals)

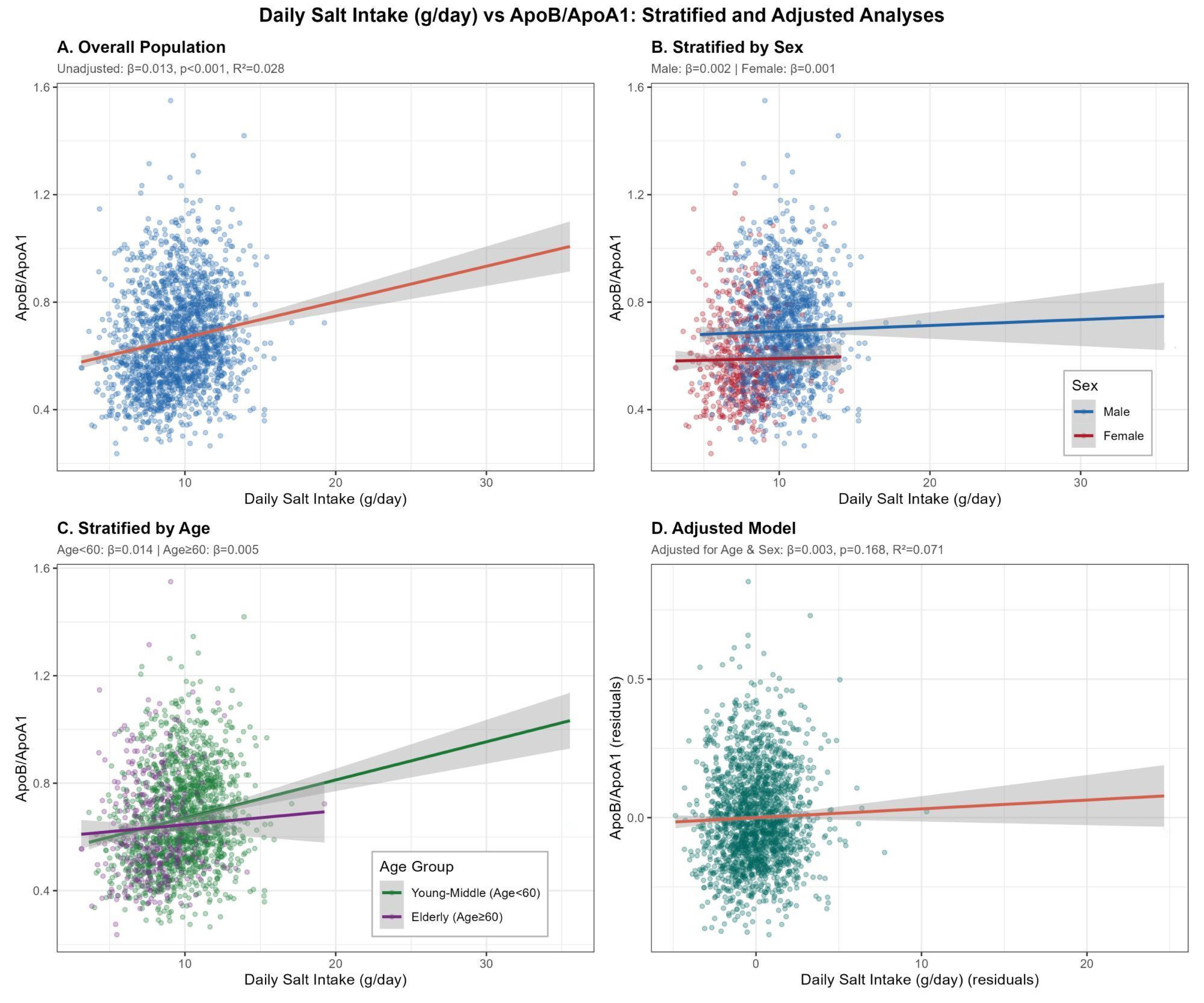
Daily Salt Intake (g/day) vs ApoB/ApoA1: Stratified and Adjusted Analyses
A. Overall Population
Unadjusted: β=0.013, p<0.001, R²=0.028
ApoB/ApoA1
Daily Salt Intake (g/day)
B. Stratified by Sex
Male: β=0.002 | Female: β=0.001
Sex
Male
Female
C. Stratified by Age
Age<60: β=0.014 | Age≥60: β=0.005
Age Group
Young-Middle (Age<60)
Elderly (Age≥60)
D. Adjusted Model
Adjusted for Age & Sex: β=0.003, p=0.168, R²=0.071
ApoB/ApoA1 (residuals)
Daily Salt Intake (g/day) (residuals)

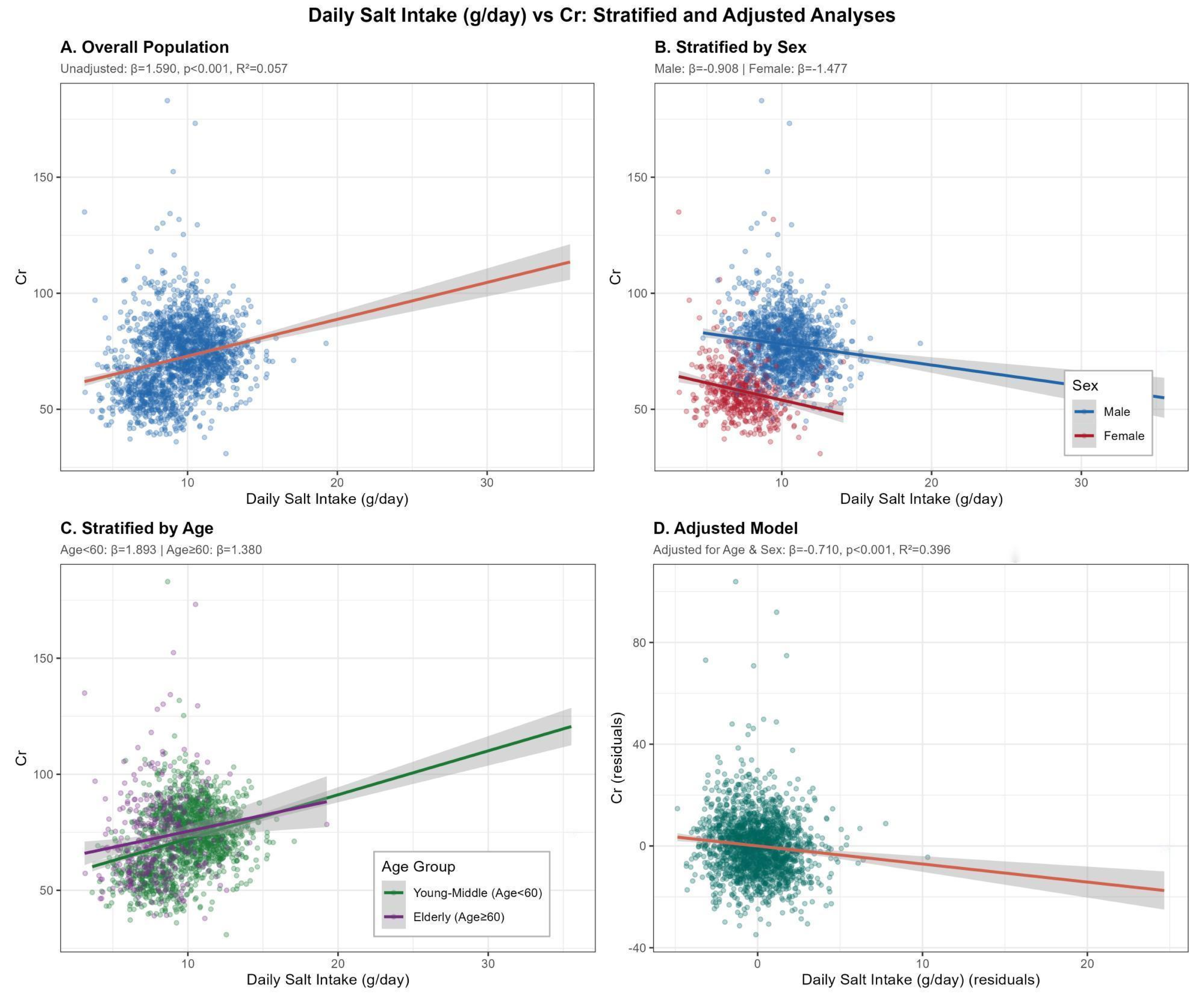

Daily Salt Intake (g/day) vs Cr: Stratified and Adjusted Analyses
A. Overall Population
Unadjusted: β=1.590, p<0.001, R²=0.057
Cr
Daily Salt Intake (g/day)
B. Stratified by Sex
Male: β=-0.908 | Female: β=-1.477
Sex
Male
Female
C. Stratified by Age
Age<60: β=1.893 | Age≥60: β=1.380
Age Group
Young-Middle (Age<60)
Elderly (Age≥60)
D. Adjusted Model
Adjusted for Age & Sex: β=-0.710, p<0.001, R²=0.396
Cr (residuals)
Daily Salt Intake (g/day) (residuals)

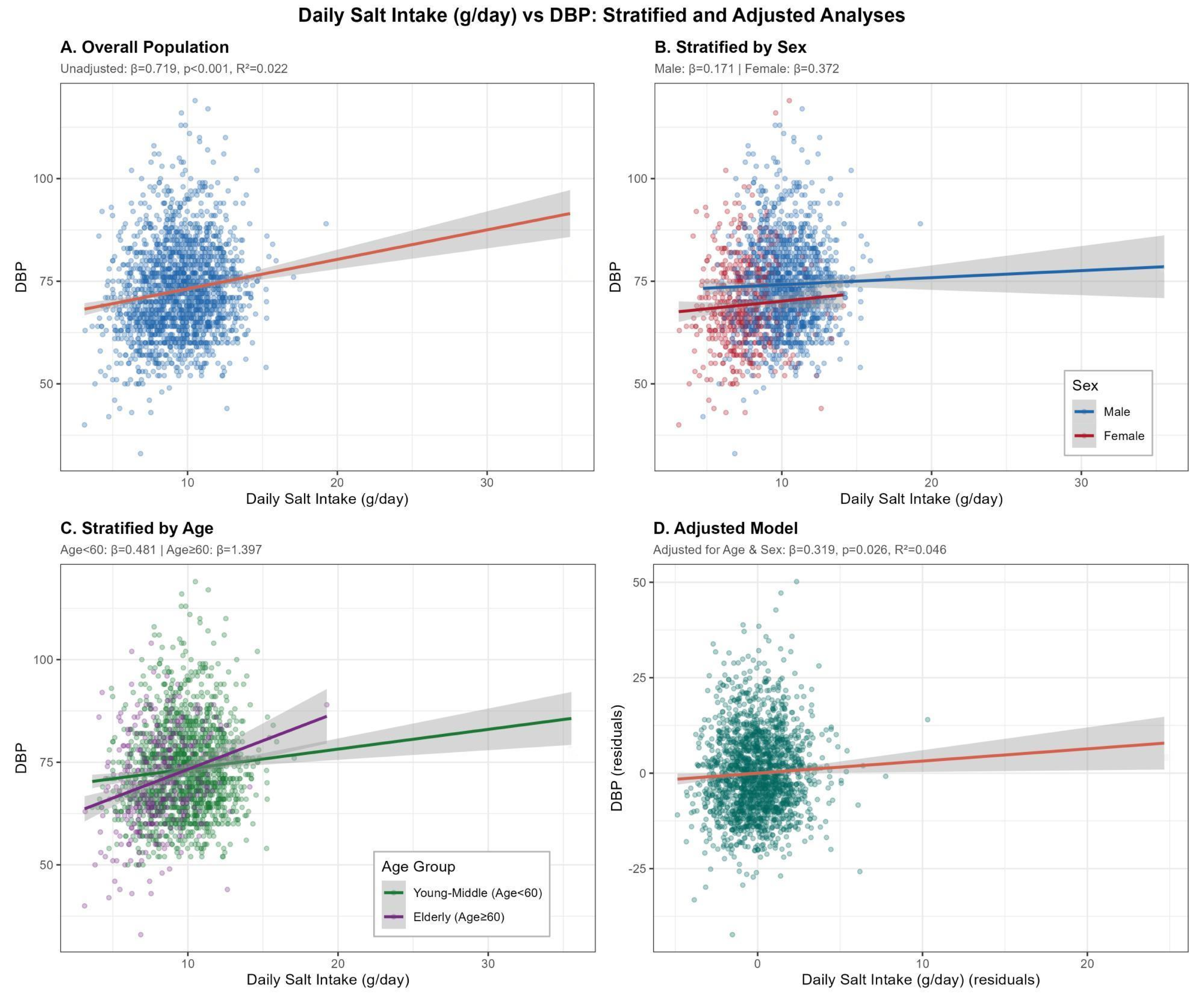
Daily Salt Intake (g/day) vs DBP: Stratified and Adjusted Analyses
A. Overall Population
Unadjusted: β=0.719, p<0.001, R²=0.022
DBP
Daily Salt Intake (g/day)
B. Stratified by Sex
Male: β=0.171 | Female: β=0.372
Sex
Male
Female
C. Stratified by Age
Age<60: β=0.481 | Age≥60: β=1.397
Age Group
Young-Middle (Age<60)
Elderly (Age≥60)
D. Adjusted Model
Adjusted for Age & Sex: β=0.319, p=0.026, R²=0.046
DBP (residuals)
Daily Salt Intake (g/day) (residuals)

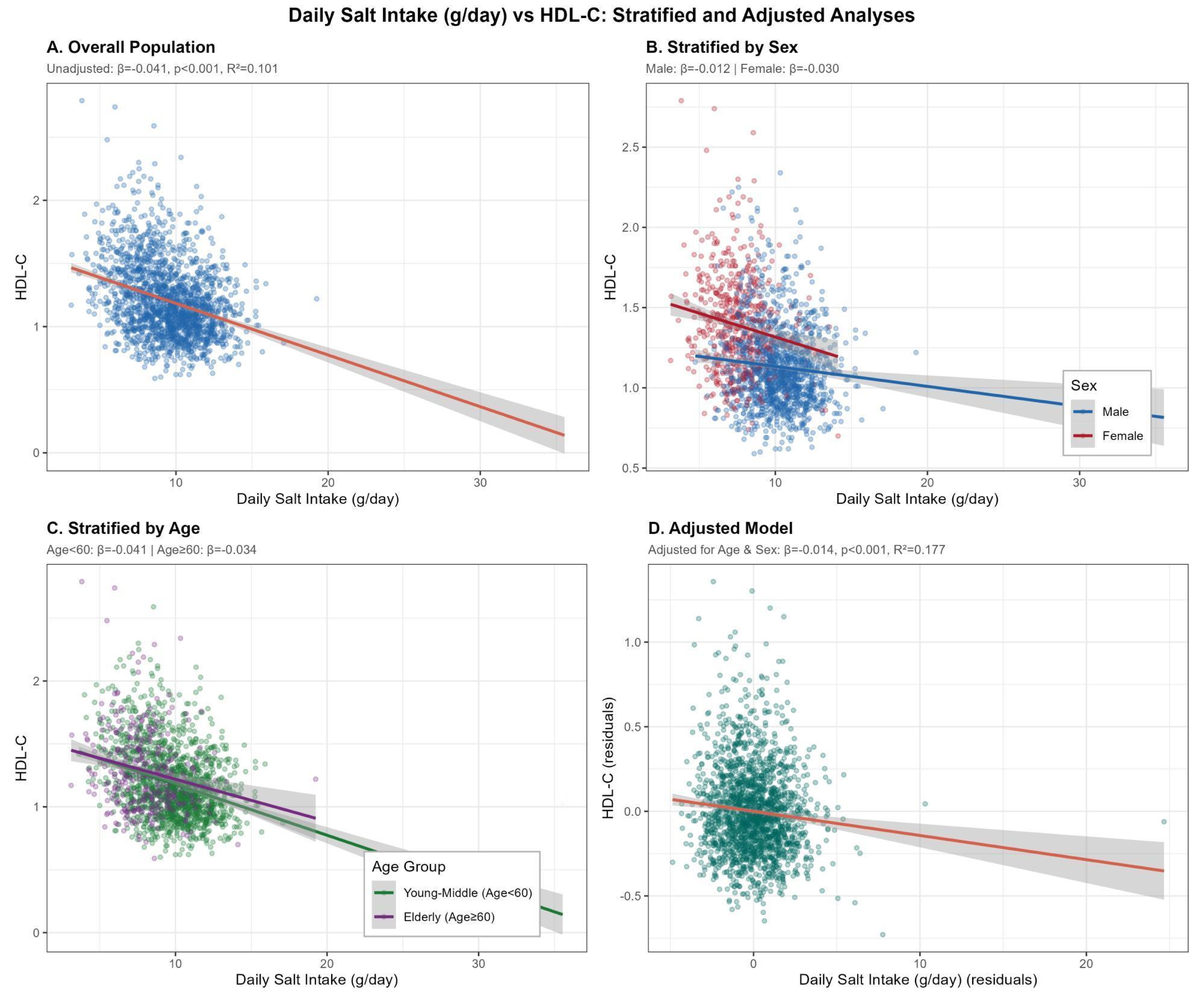
Daily Salt Intake (g/day) vs HDL-C: Stratified and Adjusted Analyses
A. Overall Population
Unadjusted: β=-0.041, p<0.001, R²=0.101
HDL-C
Daily Salt Intake (g/day)
B. Stratified by Sex
Male: β=-0.012 | Female: β=-0.030
Sex
Male
Female
C. Stratified by Age
Age<60: β=-0.041 | Age≥60: β=-0.034
Age Group
Young-Middle (Age<60)
Elderly (Age≥60)
D. Adjusted Model
Adjusted for Age & Sex: β=-0.014, p<0.001, R²=0.177
HDL-C (residuals)
Daily Salt Intake (g/day) (residuals)

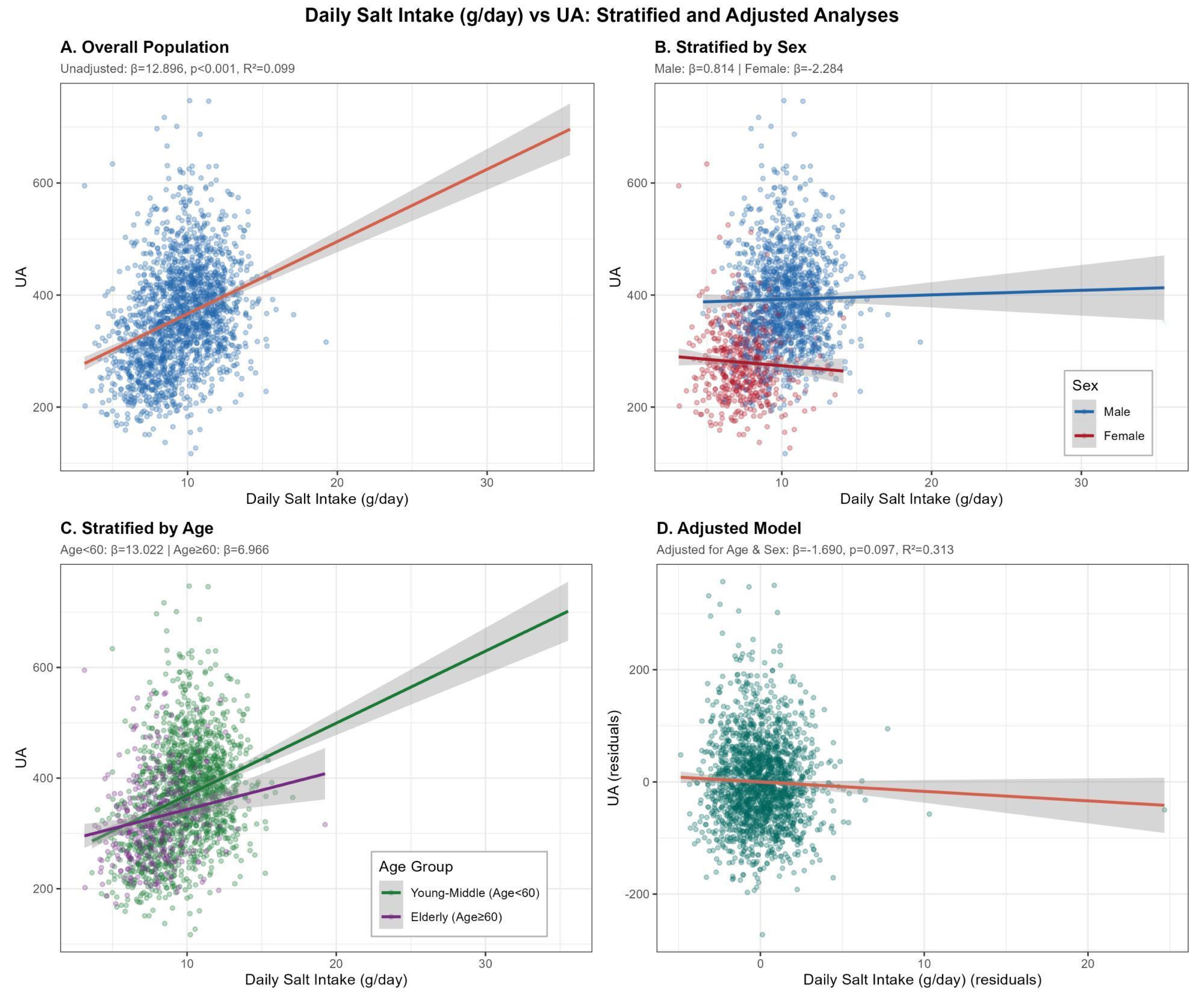

Daily Salt Intake (g/day) vs UA: Stratified and Adjusted Analyses
A. Overall Population
Unadjusted: β=12.896, p<0.001, R²=0.099
UA
Daily Salt Intake (g/day)
B. Stratified by Sex
Male: β=0.814 | Female: β=-2.284
Sex
Male
Female
C. Stratified by Age
Age<60: β=13.022 | Age≥60: β=6.966
Age Group
Young-Middle (Age<60)
Elderly (Age≥60)
D. Adjusted Model
Adjusted for Age & Sex: β=-1.690, p=0.097, R²=0.313
UA (residuals)
Daily Salt Intake (g/day) (residuals)
